# Epigenetic biomarkers in inflammatory bowel diseases—computational challenges and opportunities

**DOI:** 10.1093/ecco-jcc/jjag022

**Published:** 2026-05-19

**Authors:** Seokjun Lee, Jaesub Park, Hyun Chang Lee, Xingze Xu, Ellie Slater, Marco Gasparetto, Namshik Han, Matthias Zilbauer

**Affiliations:** Cambridge Stem Cell Institute, University of Cambridge, Cambridge, United Kingdom; Milner Therapeutics Institute, University of Cambridge, Cambridge, United Kingdom; Department of Applied Mathematics and Theoretical Physics, Cambridge Centre for AI in Medicine, University of Cambridge, Cambridge, United Kingdom; Cambridge Stem Cell Institute, University of Cambridge, Cambridge, United Kingdom; Milner Therapeutics Institute, University of Cambridge, Cambridge, United Kingdom; Department of Applied Mathematics and Theoretical Physics, Cambridge Centre for AI in Medicine, University of Cambridge, Cambridge, United Kingdom; Cambridge Stem Cell Institute, University of Cambridge, Cambridge, United Kingdom; Milner Therapeutics Institute, University of Cambridge, Cambridge, United Kingdom; Department of Applied Mathematics and Theoretical Physics, Cambridge Centre for AI in Medicine, University of Cambridge, Cambridge, United Kingdom; Cambridge Stem Cell Institute, University of Cambridge, Cambridge, United Kingdom; Milner Therapeutics Institute, University of Cambridge, Cambridge, United Kingdom; Department of Applied Mathematics and Theoretical Physics, Cambridge Centre for AI in Medicine, University of Cambridge, Cambridge, United Kingdom; Cambridge Stem Cell Institute, University of Cambridge, Cambridge, United Kingdom; Department of Paediatrics Gastroenterology, Norfolk and Norwich University Hospitals, Jenny Lind Children’s Hospital, Norwich, Norfolk, United Kingdom; Norwich Medical School, Faculty of Medicine and Health Science, University of East Anglia, Norwich, Norfolk, United Kingdom; Cambridge Stem Cell Institute, University of Cambridge, Cambridge, United Kingdom; Milner Therapeutics Institute, University of Cambridge, Cambridge, United Kingdom; Department of Applied Mathematics and Theoretical Physics, Cambridge Centre for AI in Medicine, University of Cambridge, Cambridge, United Kingdom; Department of Quantum Information, Institute for Convergence Research and Education in Advanced Technology and Engineering, Yonsei University, Seoul, Republic of Korea; Department of Nano Biomedical Engineering (NanoBME), Advanced Science Institute, Yonsei University, Seoul, Republic of Korea; Center for Nanomedicine, Institute for Basic Science (IBS), Seoul, Republic of Korea; Cambridge Stem Cell Institute, University of Cambridge, Cambridge, United Kingdom; Department of Paediatrics, University of Cambridge, Cambridge, United Kingdom; Department of Paediatric Gastroenterology, Hepatology and Nutrition, Cambridge University Hospitals (CUH), Addenbrooke’s, Cambridge, United Kingdom

**Keywords:** inflammatory bowel disease, epigenetic biomarkers, DNA methylation, precision medicine, multi-omics integration, machine learning

## Abstract

Inflammatory bowel diseases (IBD) remain a therapeutic challenge due to their heterogeneous nature and the absence of clinically actionable biomarkers to guide precision treatment. While multi-omics studies have advanced our understanding of the disease, meaningful translation into practice has been hindered by lack of validation and a need to move from association to a more generalizable signal. Epigenetics, particularly DNA methylation, offers a promising lens to capture disease-relevant regulatory states that reflect both genetic predisposition and environmental influences. However, the path to clinical adoption has been limited by persistent computational hurdles. Here we synthesize the current landscape of IBD biomarker discovery and highlight the conceptual advantages of epigenetic signatures. We outline the main obstacles that have limited clinical translation to date, including data heterogeneity, batch effects, and the challenge of distinguishing functional “driver” from non-functional “passenger” epigenetic changes. We then discuss how recent advances in computational methodology—spanning data harmonization, integrative modeling, and interpretable machine learning—can help bridge the gap between complex datasets and reliable, deployable biomarkers. Finally, we propose a forward-looking roadmap for study design and validation aimed at moving the field toward routine clinical implementation, thereby realizing the full potential of epigenetics in IBD.

## 1. Introduction

The number of medical treatments available to physicians managing patients with inflammatory bowel disease (IBD) continues to grow rapidly. Most, if not all, of these treatments target various aspects of the immune system. However, existing treatments are effective in only up to 60% of cases, with many drugs failing to benefit the majority of IBD patients.^1^ Furthermore, even treatments that initially induce a strong response, including those leading to deep remission, often fail to prevent chronic inflammation from relapsing.^2^

A critical challenge in IBD treatment is the absence of reliable biomarkers that can predict which patients will benefit from a particular drug at a specific point in their disease course. Without such markers, and as the number of available therapies continues to grow, clinicians face increasing difficulties in selecting the most appropriate and beneficial treatment strategies for individual patients.^3^ This highlights the urgent need for clinical biomarkers that can guide evidence-based decisions regarding both medical and non-medical treatment options in IBD.

One fundamental shortcoming of current treatments is the lack of evidence that the specific mechanisms or pathways targeted by these drugs are contributing to chronic intestinal inflammation in individual patients.^4^^,^^5^ The significant variation in phenotypes and disease behavior among patients classified under the same condition suggests strongly that multiple, distinct molecular mechanisms may contribute to disease development and persistence in IBD. This variability probably explains why efficacy can differ substantially even among patients with apparently similar clinical phenotypes.

Identifying patient-specific molecular mechanisms is therefore expected to facilitate the development of more targeted therapies and to provide guidance on the optimal use of existing drugs. Even in the absence of clear disease-causing mechanisms, molecular profiling of disease-relevant tissues or cell types offers the opportunity to discover patterns that vary among patients with similar clinical phenotypes. Such molecular classifiers can form the basis for clinical biomarkers, provided they are shown to be robustly associated with disease definition, long-term disease outcomes, or responses to specific treatments.^6^

However, developing reliable biomarkers has proven complex and challenging, as decades of research and several high-profile failures have demonstrated. Key obstacles include the need for sufficiently large patient cohorts with long-term follow-up, careful selection of tissues and molecular read-outs, and the analytical challenges associated with high-dimensional, heterogeneous data.^3^ The last of these challenges is becoming increasingly acute as multi-omics and digital health data continue to expand at scale. While rapid advances in machine learning and artificial intelligence (AI) offer powerful tools to interrogate these datasets, their clinical impact remains fundamentally constrained by unresolved computational and analytical bottlenecks.

Among the molecular mechanisms being increasingly investigated in IBD are epigenetic modifications, such as DNA methylation (DNAm).^7^ Epigenetic mechanisms are known to regulate gene transcription and cellular function, with alterations increasingly linked to the development and chronic inflammation seen in IBD. DNAm, one of the most stable epigenetic marks,^8^ has garnered particular interest due to its potential as a biomarker. The existence of robust protocols for genome-wide profiling has led to a growing number of studies exploring the use of DNA methylation profiles as diagnostic and prognostic biomarkers in IBD.

However, despite the excitement in this area of research, substantial challenges remain. In this review, we provide a concise overview of the existing evidence for the use of DNAm as a clinical biomarker in IBD. While biomarker research spans multiple domains, we focus primarily on predictive and prognostic biomarkers, where treatment decision-making and trial stratification needs are most pressing. We then highlight the computational challenges that currently limit translation, particularly as AI-enabled methods become increasingly common. Finally, by introducing computational approaches that bridge clinical intent and analytical implementation, we aim to support more effective communication between clinicians and computational scientists, and provide practical recommendations and future opportunities based on our experience in this field.

## 2. Biomarkers in IBD

Biomarkers are essential tools in modern medicine, serving as measurable indicators that associate with clinical outcomes, such as hospitalization, relapse, or treatment response.^9^ Unlike clinical outcome metrics that reflect how patients feel and function, biomarkers act as surrogate measures to stratify patients based on outcomes and inform intervention strategies.^10^ Broadly, biomarkers can be categorized into diagnostic (to identify disease presence), predictive (to forecast response to therapy), and prognostic (to predict disease course).^11^ In keeping with the pressing translational focus, here we review diagnostic biomarkers briefly for context, while placing primary emphasis on prognostic and predictive biomarkers that support patient stratification and treatment selection.

**Figure 1.**
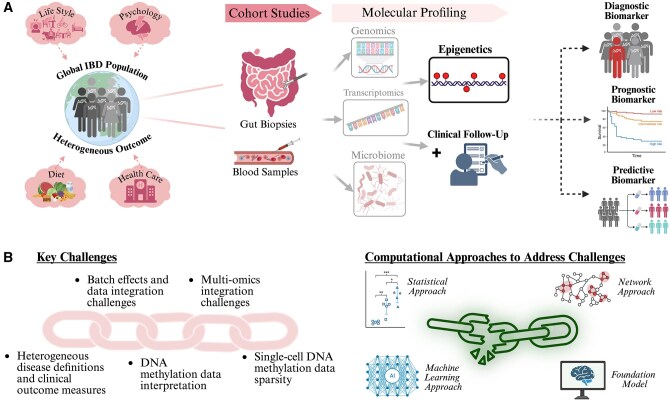
(A) Overview of inflammatory bowel disease (IBD) cohort studies that profile patients’ molecular signatures and correlate them with clinical outcomes to identify diagnostic biomarkers for IBD, prognostic biomarkers for disease progression, and predictive biomarkers for treatment response. (B) Summary of the major challenges hindering IBD biomarker development and computational strategies that offer potential solutions.

An ideal biomarker must be tailored to the disease in question, offering high specificity, sensitivity, cost-efficiency, and practicality for clinical application.^12^ Biomarkers exist in various forms, spanning simple clinical measurements to complex molecular multi-omics signatures. However, despite decades of research, no molecular biomarkers for patient stratification are clinically implemented beyond standard inflammatory markers.^13^ In this section, we review the current efforts in IBD biomarker development across different molecular modalities. We summarize findings, and highlight limitations and key challenges that could be addressed through computational approaches (Figure 1). Rather than providing an exhaustive survey of non-epigenetic biomarkers, we focus on representative landmark studies and recurrent translational barriers that motivate the need for epigenetic biomarkers.

### 2.1. Clinical and serological biomarkers

Clinical and serological biomarkers are among the earliest tools developed for disease stratification and management. Their routine use in clinical practice allows for the study of large patient cohorts in a cost-effective and non-invasive manner, offering relatively easy applicability in prospective studies.

The RISK cohort, a prospective pediatric study, identified age at diagnosis, African-American ethnicity, and isolated ileal disease location as predictors of disease progression, defined by the need for biologics or surgery.^14^ It also highlighted fibrogenesis in patients unresponsive to anti-tumor necrosis factor α (anti-TNFα) therapy, with the model showing a high negative predictive value (95%) but a limited positive predictive value (14%). Another prospective STORI cohort demonstrated that serum inflammatory markers, including C-reactive protein (CRP) and interleukin-6 (IL-6), were associated with relapse risk during infliximab withdrawal.^15^^,^^16^ However, the findings were complicated by inconsistent relapse definitions, and failed validation in the independent SPARE cohort.^17^ Similarly, the PROTECT cohort study associated increased hemoglobin and lower disease severity at diagnosis with treatment escalation in pediatric ulcerative colitis (UC), though long follow-ups and attrition limited assessment of long-term outcomes.^18^

Despite their potential ease of clinical application, these biomarkers represent systemic inflammation, hence lacking tissue and disease specificity, primarily reflecting late-stage processes.^19^ Furthermore, they often fail to provide the specificity and sensitivity needed to detect early, localized changes, making them unsuitable for assessing patient responses to interventions. Overall, clinical and serological data alone are insufficient for patient stratification and must be integrated with other biological signatures to inform clinical decisions.

### 2.2. Multi-omics biomarkers

Molecular multi-omics data have revolutionized our understanding of disease by providing deeper insights into the molecular mechanisms underlying IBD.^13^^,^^20^ In contrast to clinical data, these biomarkers quantify molecular activity at various omics levels, such as genomics, transcriptomics, epigenomics, and microbiomics.^21^ While these approaches often require specialized sample processing and interpretation, and incur higher costs, they offer novel opportunities to uncover disease-specific patterns and interactions. Here, we review studies aimed at identifying more robust biomarkers, mainly focusing on prognostic and predictive markers, that can further support the development of actionable therapeutic targets.

#### 2.2.1. Genomic biomarkers

Genomic biomarkers, driven by genome-wide association studies (GWAS), have been instrumental in identifying genetic loci linked to IBD susceptibility and disease progression. These biomarkers are particularly advantageous for their ability to reveal stable, heritable factors that influence disease risk across generations.

Over 200 genetic loci have been identified in IBD to date. Notably, NOD2 variants have been associated with a higher risk of surgery due to stricturing Crohn’s disease (CD) phenotype.^22^ This genetic marker highlighted pathways central to IBD pathogenesis, including epithelial barrier function and microbial defence.^23^ Similarly, a retrospective analysis of the UK IBD Genetics Consortium cohort identified FOXO3 and IGFBP1 variants, associated with indolent CD that does not require early treatment escalation.^24^ Beyond prognosis, genomic markers have informed treatment strategies. The prospective PANTS study identified increased anti-drug antibodies against anti-TNF therapy in adult patients carrying the HLA-DQA1*05 haplotype and recommended pre-treatment genetic screening to predict biologic response.^25^^,^^26^ Likewise, TPMT variants, known for their role in thiopurine metabolism, were shown to predict hematologic adverse drug reactions during thiopurine treatment.^27^

While being the most stable and heritable marker, challenges persist for their clinical application. Most IBD-associated single nucleotide polymorphisms (SNPs) are located in intergenic regions, making their biological significance and target genes difficult to resolve.^28^ Because many risk variants act in a tissue- and cell-type-specific manner, linking a given locus to the most relevant cell types or intestinal segments further complicates functional interpretation. Additionally, many discovery studies have been carried out in relatively homogeneous populations, limiting genetic diversity and hindering validation in independent ancestries. The binary nature of SNPs, combined with the low frequency of some risk alleles, also limits statistical power to detect small effect sizes.^29^ In this respect, polygenic risk scores (PRSs) offer one way to aggregate these modest effects. For example, a recent PRS for UC has been shown to predict susceptibility to immune checkpoint inhibitor-mediated colitis.^30^ However, the optimal way to deploy PRSs in routine IBD care remains unclear, and their added value beyond existing clinical predictors has not yet been firmly established. As such, although genomic biomarkers can define specific patient subgroups with distinct prognosis or treatment response, their overall generalizability and routine clinical use remain limited due to modest effect sizes, incomplete functional understanding, and the need for validation across diverse populations.

#### 2.2.2. Transcriptomics biomarkers

In recent decades, large-scale gene expression profiling has generated vast RNA sequencing data, revealing both whole-tissue or single-cell gene expression changes associated with IBD.^31^^,^^32^ Transcriptomic biomarkers offer the advantage of capturing dynamic changes in gene activity, reflecting both disease states and response to treatment.

The UK-led PROFILE trial is a key prospective, biomarker-stratified study that evaluated whether a peripheral blood CD8^+^ T-cell transcriptional signature could predict disease course and guide early treatment strategy in newly diagnosed CD.^33^^,^^34^ While promising in the discovery cohort, the PROFILE trial demonstrated the challenges of translating molecular risk stratification into clinical decision-making, as modifying treatment strategy based on the CD8^+^ T-cell transcriptional signature did not result in improved patient outcomes in the prospective trial setting.^35^ The prospective MSCCR cohort study derived molecular inflammation scores, engineered from transcriptomic profiling of intestinal biopsies and blood samples, to predict responses to infliximab and vedolizumab.^36^ These scores achieved an AUROC (area under the receiver operating characteristic curve) of 0.73, though variability across biopsy sites and demographic differences limits their consistency. Another prospective study identified 15 differentially expressed transcripts, including S100A12 and ANOS1, as biomarkers for predicting clinically active pediatric IBD.^37^ Similarly, a retrospective study observed downregulation of the TREM-1 transcript in whole blood among non-responders to anti-TNFα therapy.^38^

Despite current efforts, it has been difficult to translate transcriptional biomarkers into clinical practice. The dynamic and context-dependent nature of gene expression makes it highly variable across tissue types, regions, disease stages, and inflammatory status, complicating standardization and reproducibility. RNA degradation during sample collection and processing further limits data quality, and whole-tissue transcriptomes may obscure differences in cell type proportions.^39^ Although single-cell sequencing offers a solution, its high cost hinders scalability in clinical settings.^39^ As such, the use of transcriptomic biomarkers in IBD remains underdeveloped.

#### 2.2.3. Microbiomics biomarkers

Although the precise cause of IBD remains unknown, bacterial dysbiosis is widely recognized as a hallmark of the disease.^40-42^ Assessing gut health through the microbiome or metabolome of fecal samples has become an increasingly common, non-invasive technique for disease monitoring and biomarker discovery.^43^ Such a method reflects the complex relationship between gut health and environmental factors like diet and smoking that contribute to IBD pathogenesis.^44^^,^^45^

The prospective 1000IBD cohort study identified 12 microbial species and 16 microbial functional pathways as prognostic biomarkers for therapy intensification.^46^ While the discovery cohort demonstrated an AUROC of 0.75, the small sample size and variability in microbiome studies limited external validation. Similarly, the PRISM cohort study identified 40 microbiome compositions and functional pathways as predictive biomarkers for response to anti-integrin therapy (vedolizumab).^47^ The model achieved an AUROC of 0.87 among refractory IBD patients, while the extended sample collection period during long-term follow-up reduced statistical power. Metabolomics provides an alternative by analyzing metabolites produced by gut bacteria as indicators of dysbiosis. For instance, patients with IBD presented with reduced levels of short-chain fatty acids (SCFAs) in fecal matter, representing depletion in SCFA-producing bacteria.^48^^,^^49^ Additionally, a prospective study identified fecal bile acids as predictive biomarkers for response to anti-TNF therapy.^50^

Overall, quantifiable links between the microbiome and disease outcome remain weak. Microbiome and metabolome compositions vary greatly by diet, medications, and sampling methods, and between individuals and over time, reducing their reproducibility as predictors.^51^ Additionally fecal samples often fail to capture the regional-specific nature of the microbiome along the gastrointestinal tract.

While significant progress has been made in developing biomarkers for IBD, each modality presents unique challenges. Clinical and serological biomarkers, while accessible, lack specificity and mechanistic resolution. Multi-omics approaches offer greater potential but are often prone to high variability and require sophisticated modeling. These challenges have motivated growing interest in epigenetic biomarkers. DNAm in particular offers molecular signatures that, at specific loci, can be relatively stable over time and across sample types, while still reflecting cumulative environmental exposures such as diet, medication, and microbiome composition. In the next section, we review current efforts in epigenetic studies in IBD, with a focus on DNAm.

## 3. Epigenetics in IBD

Despite advances in understanding the genetic and immune-mediated mechanisms of IBD, reliable clinical biomarkers for patient stratification and therapeutic management remain elusive. Recent research has increasingly emphasized the importance of epigenetic markers—chemical modifications to DNA or chromatin that influence gene expression without changing the DNA sequence. Among these, DNAm has emerged as a particularly attractive biomarker candidate. DNAm profiles are mitotically heritable and highly cell-type specific, encoding an “epigenetic memory” of developmental origin and long-term environmental exposures. Large-scale methylation atlases in humans and mice have shown that patterns cluster by cell type rather than by individual, highlighting their stability within a given lineage.^8^^,^^52^^,^^53^ At the same time, DNAm patterns change systematically with age, underpinning widely used “epigenetic clock” measures of biological aging^54^ and illustrating their dynamic nature. Sustained inflammatory signaling, dietary patterns, and medications can also modify DNAm at specific loci, particularly in regulatory regions, providing a molecular record that integrates stable developmental programming with re-written signatures driven by disease-relevant exposures.^55^

DNAm markers therefore provide several practical advantages. They can be measured in a range of clinically accessible sample types (including blood, tissue biopsies, and patient-derived organoids), often display long-term stability within the same cell types, and may predict disease progression or response to therapy. In the following sections, we first review how DNAm biomarkers have already demonstrated significant clinical utility in other disease domains. We then discuss emerging evidence supporting DNAm-based biomarker discovery in IBD. Finally, we address the current challenges and limitations associated with applying DNAm biomarkers in the IBD context, setting the stage for computational solutions that may accelerate their clinical translation.

### 3.1. DNAm as successful biomarkers across other disease domains

DNAm biomarkers are well-established in the oncology field, highlighting their potential in early diagnosis. In colorectal cancer (CRC), two DNAm-based tests, Cologuard and Epi proColon, have received FDA approval. Cologuard, a multitarget stool DNA test, employs methylation biomarkers (NDRG4 and BMP3) combined with fecal immunochemical testing (FIT), achieving superior sensitivity (92.3%) for CRC detection, compared to FIT alone.^56^ Epi proColon detects SEPT9 promoter hypermethylation in plasma, achieving approximately 68% sensitivity for CRC detection, offering a non-invasive screening option.^57^ In neuro-oncology, MGMT promoter methylation serves as a predictive biomarker for therapeutic response in glioblastoma.^58^^,^^59^ The presence of MGMT methylation predicts better responsiveness and survival outcomes following alkylating chemotherapy with temozolomide, making it the standard care to guide clinical decisions.^60^ Similarly, methylation profiling of GSTP1, APC, and RASSF1 genes in ConfirmMDx tests has shown potential for prostate cancer diagnosis, achieving promising sensitivity (74.1%) and specificity (60%) in an African-American cohort.^61-63^ Beyond diagnostics, DNAm profiling has transformed central nervous system (CNS) tumor classification, with the Heidelberg classifier now incorporated into the WHO CNS4 framework as a recommended tool to support accurate tumor sub-typing.^64^

Beyond oncology, DNAm biomarkers in autoimmune diseases have revealed significant correlations with disease activity and therapeutic response. Rheumatoid arthritis patients display hypomethylation in immune-regulatory genes such as TRIM68, TNFSF11, and TNFSF13B, correlating with disease severity.^65^ Similarly, in systemic lupus erythematosus, extensive DNAm alterations, notably hypomethylation of interferon-regulated genes like IFI44L, have been strongly linked to active disease states.^66^^,^^67^ Likewise, the methylation status of key genes such as ZCCHC14 and KLF11 has been explored as a potential early indicator of type 1 diabetes, often preceding clinical symptoms.^68^ Collectively, these examples illustrate the growing importance of DNAm biomarkers, driven by their stability and detectability across diverse sample types and their ability to capture disease-specific changes.

### 3.2. DNA methylation in IBD

Recently, a growing body of evidence supports the utility of DNAm-based biomarkers for monitoring disease activity and therapeutic response in IBD. Epigenome-wide association studies (EWAS) in peripheral blood consistently reported widespread methylation changes, with the largest cohorts identifying thousands of differentially methylated CpG sites (Table 1). In contrast to many oncology settings, IBD-associated changes are predominantly hypomethylations and often map to immune-related genes. Across pediatric and adult cohorts, methylation at loci such as VMP1/MIR21, TRAF6, HLA regions, and RPS6KA2 distinguishes IBD patients from controls, indicating a robust systemic epigenetic signature.^69-71^^,^^76^ At the same time, these findings highlight the confounding impact of immune cell heterogeneity and inflammatory status on blood-based measurements.

**Table 1.** Summary of published DNA methylation studies in IBD.

Study	Cohort	Tissue	Key findings
**Adams et al. 2014^69^**	Pediatric discovery: 18 CD vs 18 controls; replication: 18 CD vs 18 controls. Adult validation: 20 CD vs 20 controls; extended: 87 CD vs 85 controls.	Pediatric: peripheral blood leukocytes. Adult: whole blood.	65 differentially methylated positions (DMPs) and 19 differentially methylated regions (DMRs) identified, predominantly hypomethylated in CD. Methylation changes were enriched near IBD/CD GWAS loci. The strongest signal was at VMP1/MIR21, with increased MIR21 expression. A simple 2-CpG blood classifier achieved AUC of up to 0.98 in the pediatric cohort.
**McDermott et al. 2016^70^**	Adult: 149 IBD (88 CD, 61 UC) vs 39 controls (PBMC). Subset: 79 active vs 70 inactive IBD. Pediatric validation: 24 IBD vs 22 controls (colonic tissue).	Adult: PBMC. Pediatric: colonic mucosa biopsies.	3196 DMPs in CD and 1481 in UC (≈45% overlap). *TIFAB* was among top hypermethylated genes; *TRAF6* was hypermethylated with reduced mRNA expression. 7 CD-specific and 2 UC-specific DMRs found, notably at *TRIM39-RPP21*. In pediatric colonic tissue, *TIFAB* and *TRAF6* showed opposite methylation patterns to blood, indicating tissue/age-specific regulation.
**Ventham et al. 2016^71^**	Adult discovery: 240 IBD (121 CD, 119 UC) vs 191 controls. Replication: 240 IBD vs 98 controls. Sub-analyses: sorted immune cells (*n* = 60); WGBS on subset (6 IBD vs 3 controls); gene expression (*n* = 68).	Adult: whole blood leukocytes (with subset analyses in sorted CD4^+^ T, CD8^+^ T, CD14^+^ monocytes).	439 genome-wide significant DMPs in IBD vs controls. Five robust DMRs were replicated (eg, *VMP1/MIR21*, *ITGB2*, *WDR8*, *TXK*). *TXK* was hypermethylated specifically in CD8^+^ T-cells with corresponding reduced gene expression. Unsupervised clustering of methylation separated IBD subgroups associated with risk of surgery/hospitalization, though this was not independent of cell composition and other covariates.
**Somineni et al. 2019^72^**	Pediatric (RISK cohort): 164 CD vs 74 controls at diagnosis; longitudinal follow-up 1-3 years.	Pediatric: peripheral blood.	1189 DMPs distinguished CD patients at diagnosis (82% hypermethylated). Methylation changes were enriched in immune/inflammatory pathways (TNF, JAK–STAT, IL-17) and correlated with clinical inflammation indices (CRP, PCDAI). Treatment drove most abnormal CpGs to revert toward healthy levels, suggesting these blood methylation changes reflect dynamic inflammatory burden rather than fixed traits. Only 3 CpGs showed any evidence of causal association with disease, and none could predict progression to stricturing complications.
**Mishra et al. 2022^73^**	Adult anti-TNF initiation cohort: 14 IBD (10 UC, 4 CD) treated with infliximab, vs 17 IBD (10 UC, 7 CD) on vedolizumab (therapy control); serial samples at baseline, 4 h, 24 h, 72 h, 2 weeks, 6 weeks, 14 weeks (multi-omics). Replication: 23 IBD on infliximab (baseline, 2 weeks, 6 weeks). External validation: 20 CD on infliximab (expression data).	Adult: longitudinal peripheral blood samples (DNA methylation at baseline, 2 weeks, 6 weeks; RNA-seq at all time-points).	∼85 700 DMPs associated with remission and ∼58 300 with non-remission after infliximab induction. Early during therapy (within 2 weeks), emergent methylation changes in 31 genes (linked to differentially expressed genes) yielded a strong blood-based predictor of 14-week remission (training AUC 1.00; validation AUC 0.88, accuracy 85%). This early-change model outperformed baseline methylation or clinical markers. Pathway analysis of therapy-responsive DMPs highlighted immune activation pathways. No robust baseline methylation signature predicted anti-TNF response, emphasizing the importance of time-point selection.
**Joustra et al. 2023^74^**	Adult longitudinal stability cohort: 46 IBD patients (36 CD, 10 UC) with paired blood samples ∼7 years apart (no major clinical changes between time-points).	Adult: peripheral blood leukocytes (paired samples ∼7 years apart).	194 391 DMPs exhibited significant methylation changes over time within individuals (“time-associated DMPs”), alongside shifts in cell-type composition. An estimated 60% of CpG sites showed poor intra-individual stability (ICC <0.5) across years, whereas ∼14% loci were highly stable (ICC ≥0.75). Stable methylation loci were enriched in genes for cell–cell signaling, adhesion, and neurogenesis. Notably, a minority of previously reported IBD-associated methylation sites remained stable over time (22 CD-associated, 11 UC-associated, 24 IBD-general loci), underscoring that many blood DNAm markers of IBD are temporally dynamic.
**Lin et al. 2024^75^**	Adult (PANTS study): 385 anti-TNF-naïve IBD patients (198 on infliximab, 187 on adalimumab); blood collected at baseline and weeks 14, 30, and 54 of therapy.	Adult: peripheral blood.	4999 DMPs after anti-TNF therapy exposure (infliximab/adalimumab), with enrichment in immune system processes such as JAK–STAT signaling. While baseline methylation profiles had limited ability to predict primary non-response, they were more effective in predicting drug pharmacokinetics (anti-TNF drug concentrations at week 14). This suggests DNAm may inform dose optimization even if direct response prediction is modest.
**Noble et al. 2025^76^**	Pediatric multi-cohort analysis: UK1—36 CD vs 36 controls; UK2—86 IBD (33 CD, 31 UC, 22 IBD-U) vs 30 controls; UK3—90 IBD (60 CD, 30 UC/IBD-U) + parents (trios); external validation in RISK cohort.	Pediatric: peripheral blood.	384 DMPs identified in pediatric IBD, with top hits at *ZBTB16* and *IFNAR1*. A 4-CpG blood methylation classifier (*RPS6KA2*, *VMP1*, *CFI*, *ARHGEF3*) distinguished IBD patients from controls with high accuracy (AUC ∼0.91 in UK2, ∼0.93 in RISK validation). Additionally, children with IBD showed significant epigenetic age acceleration at diagnosis (median + 3.5 years vs chronological age), highlighting the systemic impact of pediatric IBD.
**Joustra et al. 2025^77^**	Adult (EPIC-CD study): Discovery—183 IBD patients starting biologics (57 on adalimumab, 64 vedolizumab, 62 ustekinumab) from Amsterdam; Validation—90 IBD patients (32 adalimumab, 25 vedolizumab, 33 ustekinumab) from Oxford.	Adult: peripheral blood (pre-treatment samples).	Identified distinct blood methylation signatures predictive of therapy response for each biologic: 18 CpGs for adalimumab, 25 for vedolizumab, 68 for ustekinumab. In the discovery cohort, methylation-based models achieved promising performance (AUC 0.86-0.89) for vedolizumab and ustekinumab response, and these generalized moderately in validation (AUC ∼0.75). The vedolizumab/ustekinumab models improved positive response probability by 20%-24% over current clinical predictors. Notably, predictive accuracy declined in patients previously exposed to anti-TNF, suggesting therapy-specific methylation patterns.
**Harris et al. 2014^78^**	Pediatric: 14 IBD (10 CD, 4 UC) vs 10 controls; Validation—10 IBD vs 12 controls; also 2 UC patients re-biopsied in remission.	Pediatric: colonic mucosal biopsies.	3365 DMRs distinguished treatment-naïve UC from controls, while 182 DMRs distinguished CD from controls. In UC, methylation changes were correlated with transcriptional changes: ∼120 differentially expressed genes overlapped UC DMRs, enriched for immune response and antigen presentation functions. Strikingly, the colonic mucosal methylation profile in active UC reverted to a normal-like state in remission (follow-up biopsies).
**Howell et al. 2018^79^**	Pediatric: 66 IBD (43 CD, 23 UC) vs 30 controls; total 236 intestinal biopsies from terminal ileum (TI), ascending colon (AC), sigmoid colon (SC). Longitudinal subset: repeat endoscopies in 23 patients (∼1 year apart).	Pediatric: purified intestinal epithelial cells (IECs) isolated from mucosal biopsies; subset of patient-derived colonic organoids.	Genome-wide methylation of IECs revealed gut segment-specific patterns in IBD. In the sigmoid colon, an 11-CpG methylation panel distinguished IBD from controls with AUC 0.94, while a 9-CpG panel in terminal ileum IECs distinguished CD from UC (AUC 0.92). Many disease-associated IEC methylation marks were stable over ∼1 year and persisted *ex vivo* in cultured organoids. These findings suggest epithelial methylation differences are inherent to disease subtype and gut location, and not merely reactive to transient inflammation.
**Agliata et al. 2020^80^**	Pediatric & adult: 285 intestinal biopsies (204 IBD patients vs 81 controls) from multiple segments (terminal ileum, ascending colon, sigmoid colon).	Pediatric & adult: purified intestinal epithelial cells from mucosal biopsies.	4205 significant DMPs identified in IECs of IBD patients, the majority showing hypermethylation. IBD-associated DMPs were non-randomly distributed in the genome—depleted in CpG islands and enriched in open sea regions—and were enriched in pathways related to TGF-β signaling and hemostasis. Notably, methylation changes in IECs were located significantly closer to IBD GWAS loci than expected by chance, supporting a model whereby genetic risk factors and inflammatory environmental exposures both shape the gut epithelial methylome in IBD.
**Li et al. 2021^81^**	Adult case-control: 7 CD patients with penetrating ileal disease vs 7 non-IBD controls. Verification: 25 CD (penetrating) vs 7 controls by pyrosequencing; comparisons also made between penetrating and non-penetrating CD.	Adult: ileal mucosal biopsies (for CD patients, from the center of the penetrating lesion and adjacent non-penetrating area).	∼5200 DMPs (∼2978 hyper- and 2222 hypomethylated) distinguished penetrating-CD lesions from healthy ileum. Additionally, ∼3237 CpGs differed between penetrating and non-penetrating CD mucosa. Pathway analysis indicated penetration-associated hypomethylation in genes for apoptosis, IL-8 production, and extracellular matrix–receptor interactions. The most pronounced CD lesion-specific change was hypomethylation at MUC1, which correlated with disease activity (*r* = −.50, *P* = .01); consistent with MUC1 upregulation in more aggressive CD.
**Denison et al. 2024^7^**	Pediatric: 95 IBD (72 CD, 23 UC) vs 73 controls; total 312 intestinal epithelial organoid cultures derived from biopsies (duodenum, terminal ileum, sigmoid colon).	Pediatric: intestinal epithelial organoids from DUO/TI/SC mucosal biopsies.	CD-derived intestinal epithelial cells showed stable hypomethylation of MHC class I pathway genes (notably *NLRC5* and *HLA* loci) in the TI and SC, but not in DUO. This MHC-I signature was associated with increased gene expression of antigen presentation machinery and correlated with more severe phenotypes (perianal disease and need for earlier therapy escalation). From these data, a 28-CpG methylation risk score was derived, which achieved AUC ∼0.72 for predicting aggressive disease course in pediatric CD. This highlights the prognostic potential of gut-specific DNAm patterns for disease severity.

Abbreviations: IBD, inflammatory bowel disease; CD, Crohn’s disease; UC, ulcerative colitis; IBD-U, inflammatory bowel disease unclassified; DMP, differentially methylated position; DMR, differentially methylated region; DNAm, DNA methylation; GWAS, genome-wide association study; PBMC, peripheral blood mononuclear cells; WGBS, whole-genome bisulfite sequencing; RNA-seq, RNA sequencing; TNF, tumour necrosis factor; AUC, area under the curve; ICC, intraclass correlation coefficient; CRP, C-reactive protein; PCDAI, Paediatric Crohn’s Disease Activity Index; TI, terminal ileum; AC, ascending colon; SC, sigmoid colon; DUO, duodenum; IECs, intestinal epithelial cells; CpG, cytosine–phosphate–guanine dinucleotide; MHC, major histocompatibility complex; JAK–STAT, Janus kinase–signal transducer and activator of transcription; IL-17, interleukin-17; TGF-β, transforming growth factor beta.

Longitudinal blood studies show that much of this signal is dynamic rather than a fixed patient trait. In pediatric CD, methylation signatures present at diagnosis largely normalize after treatment-induced remission and closely track CRP and disease activity indices, but do not reliably predict later complications.^72^ In adult IBD, the majority of CpG sites (∼60%) exhibit poor intra-individual stability over 7 years, with only a minority remaining highly stable.^74^ These observations emphasize that the timing of sampling is critical: a single blood draw may mainly capture current inflammatory burden, treatment, and shift in cellular composition, rather than long-term prognosis.

Despite this, recent work has strengthened the clinical potential of blood DNAm, especially for predicting therapeutic response. In the EPIC-CD study, pre-treatment methylation panels were developed to predict response to biologic therapies.^77^ Models for vedolizumab and ustekinumab achieved promising discrimination in the discovery cohort (area under the curve [AUC] ∼0.87-0.89) and moderate performance in independent validation (AUC ∼0.75), improving the post-test probability of response compared with standard clinical predictors. Building on such findings, a multi-center trial (OMICROHN) is now under way for the first time to test a clinical blood DNAm assay guiding biologic choice in CD. The outcome of this trial will be pivotal in determining whether DNAm biomarkers can move from associative research to improving prospective treatment decisions. In contrast, an adalimumab signature was not validated, underlining that predictive DNAm patterns may be therapy-specific and influenced by prior drug exposure. Complementary longitudinal work during infliximab induction has shown that early on-treatment methylation changes (within the first 2 weeks) can accurately predict remission and optimal drug dosage at 14 weeks, whereas baseline methylation alone performs poorly.^73^^,^^75^ Taken together, these studies suggest that blood DNAm offers a feasible route to non-invasive prediction of biologic response, provided that disease context and sampling schedule are carefully considered.

While blood-based assays capture systemic immune activation, the intestinal mucosa is the primary disease site, and tissue DNAm may more directly reflect local pathology. Mucosal profiling in pediatric IBD has revealed thousands of differentially methylated regions in active UC.^78^ Many UC-associated methylation changes in colonic biopsies are accompanied by concordant gene expression differences in immune pathways and revert to a near-normal state with clinical remission, indicating that they are largely inflammation-driven. By contrast, methylation patterns in purified intestinal epithelial cells (IECs) show more stable, region- and disease-specific features. IEC-focused studies have identified segment-specific signatures in terminal ileum versus colon that discriminate IBD subtypes with high accuracy, and many of these markers are stable over time and retained in patient-derived organoids.^79^^,^^82^ Larger epithelial datasets further show that disease-associated CpGs are enriched in distal “open sea” regions and lie closer than expected to IBD GWAS loci, pointing to convergence between genetic risk and epithelial epigenetic remodeling.^80^

Mucosal DNAm profiling has also yielded candidate prognostic markers for disease behavior. In ileal CD, lesion-specific hypomethylation of genes such as MUC1 has been associated with penetrating complications and higher disease activity, suggesting that tissue methylation at selected loci can mark an aggressive phenotype.^81^ Organoid-based studies in pediatric CD have identified a stable hypomethylation signature in MHC class I pathway genes, including NLRC5 and HLA loci, in ileal and colonic epithelium of patients who later develop complicated disease.^7^ This signature underpins a multi-CpG risk score with moderate accuracy (AUC ∼0.72) for predicting severe course and earlier treatment escalation and remains detectable *ex vivo*, consistent with an intrinsic epithelial program rather than a transient inflammatory imprint.

Together, these studies highlight the promise of DNAm as a versatile biomarker modality in IBD capturing both systemic inflammatory activity in blood and local tissue-level reprogramming in the gut mucosa. However, as with any emerging biomarker field, several obstacles must be addressed before DNAm-based tests can be integrated into routine clinical practice.

### 3.3. Current challenges and limitations

Despite the promising data on DNAm biomarkers, the path toward clinical translation in IBD is complicated by both general challenges in biomarker development and DNAm-specific hurdles. From a broader standpoint, IBD biomarker research is often constrained by small patient cohorts and heterogeneous disease definitions, which hamper reproducibility and validation across independent studies. Well-powered longitudinal cohorts are scarce, making it difficult to capture dynamic changes in molecular profiles over the course of disease and treatment. Moreover, clinical endpoint measures in IBD, including remission, relapse, or surgical intervention, vary widely across studies, impeding direct comparisons of biomarker performance and complicating meta-analysis. Batch effects arising from differences in sample preparation, storage, or sequencing protocols further limit data integration across centers.

On the epigenetic front, additional technical complexities arise. Current sequencing methods rely mostly on bulk tissues where cell-type composition may skew methylation signals. While single-cell DNAm sequencing holds promise for dissecting heterogeneous cell populations, such technologies are still maturing and remain expensive and low-throughput. Additionally, interpretation of intergenic methylation and CpG density-dependent regulation can be challenging, especially if the biological function of these methylation events is not clearly defined. Platform-related issues such as the balance between coverage and depth in array-based methods versus genome-wide screening technologies also influence data consistency. Finally, DNAm changes do not always correlate linearly with gene expression, highlighting the need for integrated multi-omics analyses to fully understand the functional relevance of observed methylation patterns.

Addressing these challenges will primarily require methodological advances in study design. Recently, however, advanced computational approaches have been developed and applied to tackle these issues. In the next section, we review the computational frameworks and methods that have been employed to overcome these obstacles, with an emphasis on how they can accelerate the development of clinically actionable DNAm biomarkers for IBD.

## 4. Computational approaches for the development of biomarkers

**Figure 2.**
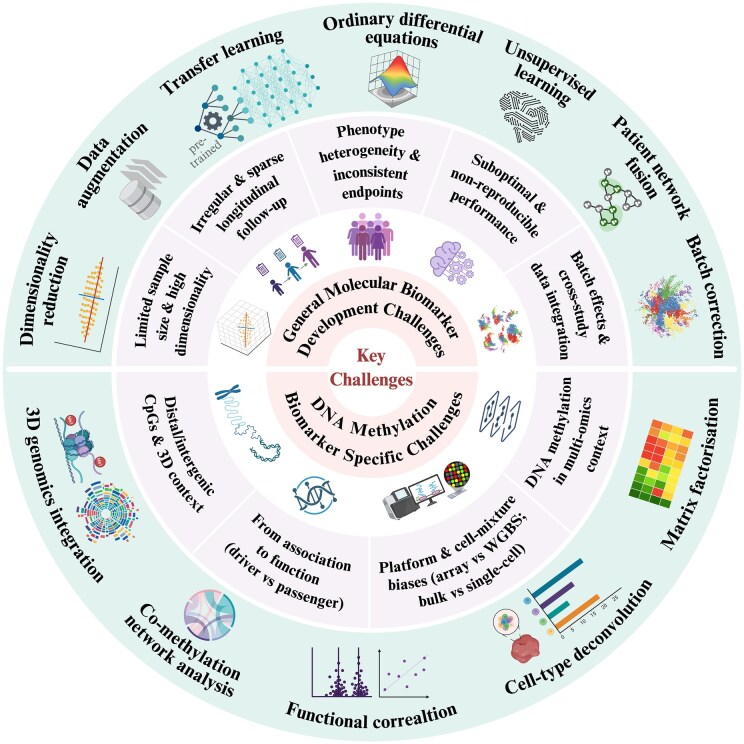
Overview of the major challenges hindering inflammatory bowel disease (IBD) biomarker development and computational approaches that offer potential solutions. The top panel summarizes general, cross-omics challenges and the corresponding computational strategies outlined in section 4.1. The bottom panel highlights DNA methylation-specific limitations and the suggested solutions described in section 4.2. WGBS, whole-genome bisulfite sequencing.

In recent years, the development of robust clinical biomarkers has been accelerated by advances in computational and data-driven methods. For IBD, these approaches are not an end in themselves but a means to solve very concrete problems: how to design adequately powered trials in small, heterogeneous patient populations; how to distil reproducible, mechanistically informative signatures from noisy multi-omics data; and how to build models that can genuinely assist treatment selection at the bedside. New methods have emerged to address these challenges at different stages of the biomarker discovery pipeline, from feature selection and patient stratification to outcome prediction and external validation (Figure 2).

Traditional statistical approaches remain a foundation of biomarker research, particularly when datasets are of moderate size. Methods such as differential analysis,^83^^,^^84^ linear or logistic regression,^85^ and survival modeling^86^ provide interpretable frameworks for hypothesis-driven studies and for deriving simple risk scores. However, these approaches may not fully capture the complex, high-dimensional relationships characteristic of omics data and may struggle to generalize across centers and platforms without additional regularization or integration strategies.

Network-based methods and machine learning (ML) extend these capabilities. Weighted gene co-expression network analysis (WGNCA), for example, groups genes into co-regulated modules that can be linked to clinical traits and used to derive module-based biomarkers.^87^ Patient similarity networks stratify individuals into subgroups based on molecular and clinical profiles, highlighting system-level interactions relevant to disease heterogeneity.^88^^,^^89^ ML algorithms are well suited to high-dimensional settings, enabling the integration and selection of informative features from thousands of CpG sites.^90^^,^^91^ Deep learning (DL) approaches extend this capability by learning hierarchical, non-linear representations of omics data.^92^^,^^93^ These methods have already been used in IBD cohorts to derive multi-modal risk signatures and to predict treatment response, and they are increasingly being embedded into the design of prospective studies and clinical trials.

Lastly, foundation models have recently emerged as a potential game changer. Borrowed from advances in natural language processing, these approaches involve pre-training algorithms on large multi-disease datasets before fine-tuning them for IBD-specific tasks.^94^ While still nascent in the multi-omics space, they hold the promise of leveraging existing knowledge to overcome sample-size limitations, thereby making sophisticated computational models more accessible for biomarker research in IBD. In the following sections, we discuss how these computational strategies are being harnessed to address key challenges in biomarker development for IBD, both at the general multi-omics level and specifically for DNAm data.

### 4.1. General molecular biomarker development challenges—IBD and beyond

#### 4.1.1. Small sample sizes and high data dimensionality

In IBD and many other complex diseases, molecular studies often involve small patient cohorts but hundreds of thousands of measured features, often described as the “large P, small N” problem.^95^ In this setting, it is easy to identify patterns that fit the current dataset but do not hold in new patients. To reduce this risk, several families of methods are used.

First, dimensionality reduction methods such as principal component analysis (PCA), t-SNE, and UMAP compress high-dimensional omics data into a smaller number of composite variables.^96-98^ This helps to highlight broad biological patterns while suppressing random noise.^99^^,^^100^ Second, statistical resampling approaches (eg, bootstrapping, cross-validation) repeatedly re-fit models on resampled versions of the data to test how stable the selected features or biomarker scores are.^101^^,^^102^ Third, data augmentation and transfer learning help when only a few patients are available. Data augmentation generates realistic synthetic samples from existing data. For instance, simulated epigenetic datasets have been shown to improve the detection of meaningful signal changes while reducing false positives.^103^ Similarly, an application in medical imaging has produced high-quality synthetic magnetic resonance imaging scans that enhance model training and support more reliable clinical interpretation.^104^ Transfer learning allows a model that has already learned general molecular patterns from large public datasets to be adapted to much smaller, disease-specific cohorts.^52^^,^^105^^,^^106^ In practice, this means the model needs only a “few examples” from the target disease to achieve state-of-art performance. This approach helps overcome data scarcity and enables more reliable biomarker discovery in rare or hard-to-collect patient groups.

**Clinical takeaway:** By filtering out irrelevant or redundant data and testing models with different sample sets, these methods can help ensure that the identified methylation candidate biomarkers are less likely to be false positives and increase the chance to be reproduced in other IBD patient groups. This is important before spending resources on clinical validation or using candidate biomarkers for trial stratification.

#### 4.1.2. Understanding disease over time: overcoming limitations of longitudinal studies

Longitudinal cohorts are essential to understand how biomarkers change during flares, remission, and treatment. In practice, however, follow-up is often irregular, patients miss visits, and some timepoints are not sampled at all. Naively comparing a few timepoints can therefore give a misleading picture of disease trajectories.

Newer approaches address this by explicitly modeling time as a continuous variable. Methods such as MEFISTO allow patient samples collected at different clinical timepoints to be placed along a shared disease trajectory, helping to capture gradual changes in disease activity and progression over time.^107^ In parallel, imputation methods tailored to longitudinal clinical and multi-omics data, such as DeepIDA, can infer missing measurements by learning patterns from patients with more complete follow-up.^108^ This allows all patients to contribute to trajectory analyses, even when their sampling is sparse.

A complementary strategy uses ordinary differential equations to describe how biological quantities change over time. For instance, inflammation-related cell populations can be modeled as dynamic systems, so that static snapshots from biopsies or blood are mapped onto inferred disease course.^109^^,^^110^ These approaches make it possible to link isolated timepoints to an underlying trajectory of worsening or improving disease.

**Clinical takeaway:** Methods that treat time as a continuous variable and fill in missing data can allow us to make better use of imperfect follow-up. This means that, in future, clinicians may be able to use just one or a few biomarker measurements to estimate a patient’s risk of flare, like disease course or need for treatment escalation, even when real-world sampling is irregular.

#### 4.1.3. Heterogeneous disease definitions and clinical outcome measures

Complex diseases like IBD often consist of multiple heterogeneous sub-phenotypes or endotypes with different underlying biology and treatment responses. Yet, patients are often grouped using broad clinical labels (eg, “CD” vs “UC”), which can hide important molecular differences and dilute biomarker signals.

Unsupervised clustering and related approaches address this by grouping patients directly on the basis of their molecular profiles rather than pre-defined labels.^111-113^ Hierarchical clustering and more advanced methods such as similarity network fusion create patient-similarity networks from multi-omics data and identify clusters of patients who share common molecular patterns.^114^ Graph-based methods such as MOGONET go a step further by combining information across several omics layers.^115^ These data-driven clusters can then be related back to clinical features such as complication rates or treatment response, revealing more homogeneous subgroups that may benefit from different management strategies.

A second, closely related problem is the lack of standardization of clinical outcomes. Definitions of “response,” “remission,” or “treatment failure” often vary between studies, making it difficult to compare or pool biomarker results. Other fields have demonstrated that consensus outcome sets greatly improve reproducibility: for example, EuroHeart has standardized cardiovascular endpoints, and perioperative medicine has harmonized definitions of complications, with clear benefits for trial comparability.^116^^,^^117^ Similar efforts in IBD (eg, standardized endoscopic and composite endpoints, harmonized ancestry and outcome descriptors) are essential if biomarkers are to be evaluated across centers and incorporated into guidelines.^118-121^

**Clinical takeaway:** Using unsupervised, data-driven methods to group patients, together with standardized outcome definitions, can reveal clinically meaningful IBD subtypes. This may improve interpretability and facilitate comparison across centers by anchoring biomarker signals to shared disease categories with more predictable clinical trajectories and treatment responses.

#### 4.1.4. Suboptimal biomarker performance

A frequent complaint is that biomarkers discovered in one study often show limited predictive performance or fail to replicate in independent cohorts. This inconsistency arises primarily due to statistical overfitting, disease heterogeneity, and reliance on individual molecular markers that lack robust predictive power.

To address this, many groups now move from single-molecule markers to network- or module-based biomarkers. Methods such as WGCNA construct a gene co-expression network (how strongly genes correlate across patient samples) and identify modules of genes whose activity rises and falls together across patients.^87^ Module-level scores, rather than individual genes, are then related to outcomes such as disease severity or treatment response. Because these modules capture whole pathways or cellular programs, they tend to be more stable across datasets and platforms.^122-124^

In parallel, feature selection algorithms help identify a smaller, more robust set of variables that carry most of the predictive information. Approaches like recursive feature elimination have been used, for example, to prioritize microbial biomarkers from very high-dimensional microbiome data.^125^ Finally, multi-omics integration methods, including DL frameworks such as GLUE, combine methylation, transcriptomic, and other data into a joint representation of a cell or patient state.^126^^,^^127^ This can improve biomarker robustness by ensuring that signals are supported across several molecular layers.

**Clinical takeaway:** Network-based and multi-omics methods prioritize pathways and coordinated molecular programs rather than single markers, which can improve robustness across cohorts. However, these approaches mitigate, but cannot fully eliminate, technical variation, and must be paired with standardized laboratory workflows and prospective multi-center validation.

#### 4.1.5. Batch effects and data integration

Differences between laboratories, protocols, platforms, and sequencing runs introduce batch effects that can easily overshadow true biological differences. In multi-center studies and meta-analyses, failing to correct these effects can lead to spurious biomarkers that simply reflect technical variation.

A range of methods now exists to harmonize datasets before biomarker discovery. Mutual Nearest Neighbours and Harmony, originally developed for single-cell integration, align samples or cells across batches by finding those with similar molecular profiles and adjusting embeddings accordingly.^128^^,^^129^ Probabilistic modeling approaches such as scVI and more classical tools like ComBat explicitly model batch-specific technical factors alongside true biological variability.^130^^,^^131^ Lessons from single-cell atlas studies emphasize the importance of careful quality control and integration if combined datasets are to be used for downstream biomarker work.^132^

**Clinical takeaway:** Methods for correcting variations between different labs, machines, and timepoints can ensure that biomarker scores mean the same thing wherever and whenever the test is performed. This consistency is crucial for turning omics-based research assays into reliable diagnostic or stratification tools that can be used in routine clinical practice.

### 4.2. DNAm data interpretation challenges in IBD

DNAm biomarkers hold significant promise as indicators of disease state and trajectory. However, their interpretation is complicated by the complexity of methylation biology and technical limitations of current measurement platforms. DNAm is typically quantified as β-values, representing the fraction of DNA molecules methylated at a specific locus (ranging from 0 to 1). While conceptually straightforward, β-values exhibit unequal variance across their range: sites that are fully methylated (β ≈ 1) or unmethylated (β ≈ 0) tend to have lower variability, whereas intermediate values (β ≈ 0.5) often display greater variability across samples.^8^ Accurately determining the significance of β-value changes and their biological relevance at specific CpG sites requires advanced computational approaches to account for variance, normalize data, and refine interpretation, especially when longitudinal stability varies between loci.

#### 4.2.1. Interpreting genomic context and regulatory functions of CpG methylation

Assigning functional interpretation to DNAm changes depends strongly on genomic context. Historically, methylation at CpG islands within promoters has been straightforwardly linked to gene repression.^8^ However, interpreting CpG methylation in intergenic or distal regulatory regions—such as enhancers, silencers, insulators, or “open sea” regions—remains challenging. Despite their distance from known transcription start sites, methylation changes in these regions are known to impact gene expression, potentially contributing to multiple diseases.^133^^,^^134^

To address this, several enhancer-to-gene (E2G) and causal-linking frameworks have been developed. They combine DNAm with transcriptomics and chromatin conformation data (eg, Hi-C) to link methylation changes at distal regulatory elements to their most likely target genes.^135^^,^^136^ For example, the “activity-by-contact” model integrates epigenetic marks with physically chromatin contacts to identify enhancer–promoter pairs,^137^ and studies have shown that methylation changes in these regions can alter CTCF binding and three-dimensional genome architecture that plays a central role in transcriptional regulation.^28^ In parallel, methylation-adapted gene set enrichment analyses correct for uneven CpG probe distribution and probe multiplicity, allowing pathway-level interpretation that is less biased by array design.^124^^,^^138^

**Clinical takeaway:** E2G and methylation-adapted enrichment methods connect changes in DNAm in non-coding regions to specific genes and pathways. These integrative methods can allow us to make sense of CpGs that were previously considered “uninformative.” This may help turn complex data into meaningful insight about etiology and potential therapeutic targets, ultimately improving the usefulness of methylation testing in practice.

#### 4.2.2. Functional relevance of methylation changes

Not every alteration in DNAm observed in disease contexts represents a functional or causal change that can be directly translated to clinical biomarkers. Many simply reflect confounding factors such as aging, altered cell-type compositions, or secondary effects of inflammation, without directly contributing to disease progression. A critical challenge is therefore differentiating between biologically relevant “driver” methylation changes, which actively influence gene regulation or phenotypic outcomes, from non-functional “passenger” events.

Integrative computational approaches have become central in addressing this challenge by linking DNAm data to other molecular and phenotypic datasets. For instance, integrating methylation with transcriptomic profiles enables identification of methylation-driven regulatory events.^139^^,^^140^ ML models that predict clinical features or genomic alterations from DNAm can further highlight sites with strong predictive value.^141^^,^^142^ Correlation network analysis offers additional approaches to discerning meaningful methylation signatures by identifying co-methylated CpG modules. Distinct co-methylation modules linked to biologically relevant traits have been successfully characterized in multiple contexts, emphasizing their value in understanding methylation-driven processes.^7^^,^^143^

**Clinical takeaway:** Focusing on CpG sites where methylation changes directly affect gene activity or important clinical features can help identify biomarkers that are truly linked to the disease. This makes it more likely that a methylation pattern will be stable, will be biologically meaningful, and may be useful for tracking treatment responses or predicting relapses.

#### 4.2.3. Multi-omics integration with DNAm

DNAm biomarkers are valuable in isolation, but their interpretability and predictive power often improve when integrated with complementary molecular layers. Multi-omics integration, however, is inherently challenging because each data type has distinct data distributions, dynamic ranges, and sources of technical artefacts,^144^ but several frameworks have been developed to address this.

Factor analysis methods such as MOFA identify latent factors that capture shared biological variation across omics layers.^145^ In chronic lymphocytic leukemia, MOFA has been used to link coordinated changes in DNAm, gene expression, and mutations to distinct biological programs and clinical risk groups.^146^^,^^147^ Other frameworks, such as similarity network fusion, construct separate patient-similarity networks for each omics type and then fuse them into a consensus network that better reflects overall disease state.^114^^,^^148^ Approaches like IntegrAO and iCluster build on this by accommodating partially incomplete datasets and enabling joint clustering across modalities, ensuring that integrative analyses remain robust even with real-world data gaps.^149^^,^^150^ More recently, methods such as iPANDDA extend multi-omics modeling further by identifying combinations of molecular alterations that act together to drive disease, allowing therapeutic targets to be prioritized in pairs rather than as isolated markers.

**Clinical takeaway:** Combining methylation with genetics, gene expression, and other data in integrated models can link biomarker signatures directly to druggable pathways and cellular programs. This may help clinicians move from simple risk scores to practical markers that not only stratify patients but also suggest which therapies, such as anti-TNF or anti-integrin agents, are most likely to benefit a given molecular subgroup.

#### 4.2.4. Platform-related issues and limited interpretability in bulk and single-cell DNAm data

DNAm profiling traditionally employed either array-based approaches—such as the Illumina EPIC BeadChip, which assays a predefined subset of CpGs biased towards CpG-rich regions^151^—or sequencing-based approaches, like whole-genome bisulfite sequencing (WGBS),^152^^,^^153^ offering comprehensive but uneven genomic coverage. These platform differences introduce significant technical biases, complicating cross-platform comparability and biomarker discovery. For instance, methylation arrays exhibit probe-specific biases and artifacts, including false intermediate beta values arising from genomic deletions. Computational pipelines address these issues by implementing quality-control pipelines and normalization algorithms, which leverage out-of-band probe signals to identify hybridization failures and mask unreliable probes.^154^^,^^155^ In WGBS, coverage variability poses a substantial analytical challenge, as sparsely covered CpG sites yield noisy or missing data. Computational tools have been developed to mitigate this to impute methylation levels at low-coverage sites based on patterns learned from higher-quality samples.^156^^,^^157^ Through such imputation strategies, WGBS data become more robust, enabling improved cross-sample comparability and more reliable biomarker identification.

A second limitation is that bulk DNAm profiles average signals across many cell types, masking cell-type-specific changes. Single-cell DNAm sequencing (eg, scWGBS, scRRBS, and snmC-seq) has emerged to address this, but current methods remain technically challenging, expensive, and sparse. As a practical alternative, computational deconvolution methods infer cell-type contributions from bulk methylation data. Pioneering approaches, such as Houseman’s algorithm, utilize known methylation reference profiles from purified cell populations to estimate cell-type proportions.^158^^,^^159^ More recent reference-free methods simultaneously infer the number and methylation profiles of constituent cell types directly from bulk data.^160^^,^^161^ Alternatively, new methods leveraging complementary single-cell RNA-sequencing data to infer tissue-specific methylation references have identified differentially methylated regions linked to specific cellular subsets.^162^

**Clinical takeaway:** Cell-type deconvolution methods estimate how different cell types (eg, T cells or epithelial cells) contribute to overall methylation signals, without requiring complex single-cell assays. This can enable development of biomarkers focussed on clinically relevant cell populations, potentially improving the accuracy and usefulness of tests that can be performed on standard biopsy or blood samples.

## 5. Further outlook

The evolution of epigenetic biomarker research in IBD is poised to benefit from transformative advances at the intersection of computational science, multi-omics integration, and digitally enabled clinical care. While IBD remains a complex, multi-factorial condition with only limited understanding of its underlying mechanisms, fast developing computational technologies applied to multi-omics analyses offer an exciting opportunity to dissect complexity and identify new relevant correlations and/or mechanistic insights.

Future efforts will probably pivot from retrospective analyses towards real-time, dynamic monitoring of disease progression through longitudinal, high-resolution datasets. In this context, several key avenues stand out:

Moving beyond isolated molecular layers, the field is set to adapt integrative frameworks that combine epigenetic profiles with genomic, transcriptomic, proteomic, and metabolomic data. The advent of robust multi-modal data fusion techniques—ranging from advanced network analyses to probabilistic and deep learning models—will enable the construction of comprehensive disease models. These models are expected to reveal novel regulatory circuits and critical driver events, thereby enhancing our ability to predict disease trajectories and therapeutic responses.

Emerging ML paradigms, such as transfer learning, promise to overcome current limitations imposed by small cohorts and heterogeneous data sources. By leveraging pre-trained models on large-scale public datasets and incorporating privacy-preserving algorithms, these approaches can facilitate cross-institutional data sharing and collaborative research without compromising patient confidentiality. Moreover, the integration of time-series analysis methods will further capture the dynamic interplay between environmental factors and epigenetic modifications.

The convergence of digital health with molecular biomarkers offers an additional layer of opportunity. Integrating DNAm signatures with electronic health records, patient-reported outcomes, imaging, and wearable sensor data could support “digital twin” frameworks, in which computational models simulate individual patient trajectories and forecast the impact of different treatment strategies. In such a setting, DNAm biomarkers would serve not as standalone tests but as one component of a broader decision-support ecosystem.

From a practical perspective, translating DNAm biomarkers into clinical use will require several concrete steps. First, pre-analytical variables (tissue type, handling, storage) and platform-specific biases must be standardized and transparently reported. Second, candidate signatures should be evaluated using pre-specified statistical analysis plans in independent, prospectively collected cohorts, with careful attention to calibration and clinical utility relative to existing tools. Third, regulatory-grade assays, such as targeted methylation panels derived from epigenome-wide discovery studies, need to be developed. Finally, successful implementation will depend on close collaboration between clinicians, statisticians, and computational scientists to ensure that biomarker models remain interpretable, align with realistic clinical workflows, and address questions that matter to patients.

In summary, epigenetic biomarkers in IBD are transitioning from the discovery phase toward a period of systematic validation and early clinical testing. Large prospective studies such as EPIC-CD demonstrate that DNAm-based predictors of treatment response are no longer a theoretical possibility but an emerging reality. To realize their full potential, future work must combine rigorous study design, advanced computation, and thoughtful clinical integration, with the overarching goal of enabling truly personalized treatment strategies for patients with IBD.

## Data Availability

No new data were generated.

## References

[1] Bernstein CN. Treatment of IBD: where we are and where we are going. Am J Gastroenterol. 2015;110:114-126. 25488896 10.1038/ajg.2014.357

[2] Ashton JJ , Green Z, Kolimarala V, Beattie RM. Inflammatory bowel disease: long-term therapeutic challenges. Expert Rev Gastroenterol Hepatol. 2019;13:1049-1063. 31657969 10.1080/17474124.2019.1685872

[3] Denson LA , Curran M, McGovern DPB, et al Challenges in IBD research: precision medicine. Inflamm Bowel Dis. 2019;25:S31-S39. 31095701 10.1093/ibd/izz078

[4] Selin KA , Hedin CR, Villablanca EJ. Immunological networks defining the heterogeneity of inflammatory bowel diseases. J Crohns Colitis. 2021;15:1959-1973. 33959743 10.1093/ecco-jcc/jjab085 PMC8575046

[5] Kong L , Pokatayev V, Lefkovith A, et al The landscape of immune dysregulation in Crohn’s disease revealed through single-cell transcriptomic profiling in the ileum and colon. Immunity. 2023;56:444-458. e5. 36720220 10.1016/j.immuni.2023.01.002 PMC9957882

[6] Martin JC , Chang C, Boschetti G, et al Single-cell analysis of Crohn’s disease lesions identifies a pathogenic cellular module associated with resistance to anti-TNF therapy. Cell. 2019;178:1493-1508. e20. 31474370 10.1016/j.cell.2019.08.008 PMC7060942

[7] Dennison TW , Edgar RD, Payne F, et al Patient-derived organoid biobank identifies epigenetic dysregulation of intestinal epithelial MHC-I as a novel mechanism in severe Crohn’s Disease. Gut. 2024;73:1464-1477. 38857990 10.1136/gutjnl-2024-332043 PMC11347221

[8] Smith ZD , Hetzel S, Meissner A. DNA methylation in mammalian development and disease. Nat Rev Genet. 2025;26:7-30. 39134824 10.1038/s41576-024-00760-8

[9] Strimbu K , Tavel JA. What are biomarkers? Curr Opin HIV AIDS. 2010;5:463-466. 20978388 10.1097/COH.0b013e32833ed177 PMC3078627

[10] Biomarkers Definitions Working Group. Biomarkers and surrogate endpoints: preferred definitions and conceptual framework. Clin Pharmacol Ther. 2001;69:89-95. 11240971 10.1067/mcp.2001.113989

[11] Spencer EA , Dubinsky MC. Precision medicine in pediatric inflammatory bowel disease. Pediatr Clin North Am. 2021;68:1171-1190. 34736583 10.1016/j.pcl.2021.07.011

[12] Ahmad A , Imran M, Ahsan H. Biomarkers as biomedical bioindicators: approaches and techniques for the detection, analysis, and validation of novel biomarkers of diseases. Pharmaceutics. 2023;15:1630. 37376078 10.3390/pharmaceutics15061630 PMC10303887

[13] Fiocchi C. Omics and multi-omics in IBD: no integration, no breakthroughs. Int J Mol Sci. 2023;24:14912. 37834360 10.3390/ijms241914912 PMC10573814

[14] Kugathasan S , Denson LA, Walters TD, et al Prediction of complicated disease course for children newly diagnosed with Crohn’s disease: a multicentre inception cohort study. Lancet. 2017;389:1710-1718. 28259484 10.1016/S0140-6736(17)30317-3 PMC5719489

[15] Pierre N , Baiwir D, Huynh-Thu VA, et al; GETAID (Groupe d‘Etude Thérapeutique des Affections Inflammatoires du tube Digestif). Discovery of biomarker candidates associated with the risk of short-term and mid/long-term relapse after infliximab withdrawal in Crohn’s patients: a proteomics-based study. Gut. 2020;70:1450-1457. 10.1136/gutjnl-2020-322100 33106355

[16] Pierre N , Huynh-Thu VA, Marichal T, et al; GETAID (Groupe d’Etude Thérapeutique des Affections Inflammatoires du tube Digestif). Distinct blood protein profiles associated with the risk of short-term and mid/long-term clinical relapse in patients with Crohn’s disease stopping infliximab: when the remission state hides different types of residual disease activity. Gut. 2023;72:443-450. 36008101 10.1136/gutjnl-2022-327321

[17] Pierre N , Huynh-Thu VA, Baiwir D, et al; GETAID and the SPARE-Biocycle research group. External validation of serum biomarkers predicting short-term and mid/long-term relapse in patients with Crohn’s disease stopping infliximab. Gut. 2024;73:1965-1973. 39134391 10.1136/gutjnl-2024-332648

[18] Hyams JS , Davis Thomas S, Gotman N, et al Clinical and biological predictors of response to standardised paediatric colitis therapy (PROTECT): a multicentre inception cohort study. Lancet. 2019;393:1708-1720. 30935734 10.1016/S0140-6736(18)32592-3 PMC6501846

[19] Luan YY , Yao YM. The clinical significance and potential role of C-reactive protein in chronic inflammatory and neurodegenerative diseases. Front Immunol. 2018;9:1302. 29951057 10.3389/fimmu.2018.01302 PMC6008573

[20] Seyed Tabib NS , Madgwick M, Sudhakar P, Verstockt B, Korcsmaros T, Vermeire S. Big data in IBD: big progress for clinical practice. Gut. 2020;69:1520-1532. 32111636 10.1136/gutjnl-2019-320065 PMC7398484

[21] Hayes CN , Nakahara H, Ono A, Tsuge M, Oka S. From omics to multi-omics: a review of advantages and tradeoffs. Genes (Basel). 2024;15:1551. 39766818 10.3390/genes15121551 PMC11675490

[22] Alvarez-Lobos M , Arostegui JI, Sans M, et al Crohn’s disease patients carrying Nod2/CARD15 gene variants have an increased and early need for first surgery due to stricturing disease and higher rate of surgical recurrence. Ann Surg. 2005;242:693-700. 16244543 10.1097/01.sla.0000186173.14696.ea PMC1409853

[23] Sidiq T , Yoshihama S, Downs I, Kobayashi KS. Nod2: a critical regulator of ileal microbiota and Crohn’s disease. Front Immunol. 2016;7:367. 27703457 10.3389/fimmu.2016.00367 PMC5028879

[24] Lee JC , Biasci D, Roberts R, et al; UK IBD Genetics Consortium. Genome-wide association study identifies distinct genetic contributions to prognosis and susceptibility in Crohn’s disease. Nat Genet. 2017;49:262-268. 28067912 10.1038/ng.3755 PMC5730041

[25] Kennedy NA , Heap GA, Green HD, et al; UK Inflammatory Bowel Disease Pharmacogenetics Study Group. Predictors of anti-TNF treatment failure in anti-TNF-naive patients with active luminal Crohn’s disease: a prospective, multicentre, cohort study. Lancet Gastroenterol Hepatol. 2019;4:341-353. 30824404 10.1016/S2468-1253(19)30012-3

[26] Powell Doherty RD , Liao H, Satsangi JJ, Ternette N. Extended analysis identifies drug-specific association of 2 distinct HLA class II haplotypes for development of immunogenicity to adalimumab and infliximab. Gastroenterology. 2020;159:784-787. 32275970 10.1053/j.gastro.2020.03.073

[27] Coenen MJH , de Jong DJ, van Marrewijk CJ, et al; TOPIC Recruitment Team. Identification of patients with variants in TPMT and dose reduction reduces hematologic events during thiopurine treatment of inflammatory bowel disease. Gastroenterology. 2015;149:907-917 e7. 26072396 10.1053/j.gastro.2015.06.002

[28] Nasser J , Bergman DT, Fulco CP, et al Genome-wide enhancer maps link risk variants to disease genes. Nature. 2021;593:238-243. 33828297 10.1038/s41586-021-03446-x PMC9153265

[29] Park J-H , Wacholder S, Gail MH, et al Estimation of effect size distribution from genome-wide association studies and implications for future discoveries. Nat Genet. 2010;42:570-575. 20562874 10.1038/ng.610 PMC4615599

[30] Middha P , Thummalapalli R, Betti MJ, et al; Princess Margaret Lung Group. Polygenic risk score for ulcerative colitis predicts immune checkpoint inhibitor-mediated colitis. Nat Commun. 2024;15:2568. 38531883 10.1038/s41467-023-44512-4 PMC10966072

[31] Xu L , Xiao T, Xu L, Zou B, Yao W. Bulk and single-cell RNA sequencing reveal the roles of neutrophils in pediatric Crohn’s disease. Pediatr Res. 2025;98:1950-1959. 40121337 10.1038/s41390-025-03961-x

[32] Ashton JJ , Boukas K, Davies J, et al Ileal transcriptomic analysis in paediatric Crohn’s disease reveals IL17- and NOD-signalling expression signatures in treatment-naive patients and identifies epithelial cells driving differentially expressed genes. J Crohns Colitis. 2021;15:774-786. 33232439 10.1093/ecco-jcc/jjaa236 PMC8095388

[33] Lee JC , Lyons PA, McKinney EF, et al Gene expression profiling of CD8+ T cells predicts prognosis in patients with Crohn disease and ulcerative colitis. J Clin Invest. 2011;121:4170-4179. 21946256 10.1172/JCI59255 PMC3196314

[34] Parkes M , Noor NM, Dowling F, et al PRedicting Outcomes For Crohn’s dIsease using a moLecular biomarkEr (PROFILE): protocol for a multicentre, randomised, biomarker-stratified trial. BMJ Open. 2018;8:e026767. 10.1136/bmjopen-2018-026767 PMC6286485 30523133

[35] Noor NM , Lee JC, Bond S, et al; PROFILE Study Group. A biomarker-stratified comparison of top-down versus accelerated step-up treatment strategies for patients with newly diagnosed Crohn’s disease (PROFILE): a multicentre, open-label randomised controlled trial. Lancet Gastroenterol Hepatol. 2024;9:415-427. 38402895 10.1016/S2468-1253(24)00034-7 PMC11001594

[36] Argmann C , Hou R, Ungaro RC, et al Biopsy and blood-based molecular biomarker of inflammation in IBD. Gut. 2023;72:1271-1287. 36109152 10.1136/gutjnl-2021-326451 PMC10014487

[37] Ostrowski J , Dabrowska M, Lazowska I, et al Redefining the practical utility of blood transcriptome biomarkers in inflammatory bowel diseases. J Crohns Colitis. 2019;13:626-633. 30541017 10.1093/ecco-jcc/jjy205 PMC6486489

[38] Gaujoux R , Starosvetsky E, Maimon N, et al; Israeli IBD research Network (IIRN). Cell-centred meta-analysis reveals baseline predictors of anti-TNFalpha non-response in biopsy and blood of patients with IBD. Gut. 2019;68:604-614. 29618496 10.1136/gutjnl-2017-315494 PMC6580771

[39] Li X , Wang CY. From bulk, single-cell to spatial RNA sequencing. Int J Oral Sci. 2021;13:36. 34782601 10.1038/s41368-021-00146-0 PMC8593179

[40] Franzosa EA , Sirota-Madi A, Avila-Pacheco J, et al Gut microbiome structure and metabolic activity in inflammatory bowel disease. Nat Microbiol. 2019;4:293-305. 30531976 10.1038/s41564-018-0306-4 PMC6342642

[41] Glassner KL , Abraham BP, Quigley EMM. The microbiome and inflammatory bowel disease. J Allergy Clin Immunol. 2020;145:16-27. 31910984 10.1016/j.jaci.2019.11.003

[42] Halfvarson J , Brislawn CJ, Lamendella R, et al Dynamics of the human gut microbiome in inflammatory bowel disease. Nat Microbiol. 2017;2:17004. 28191884 10.1038/nmicrobiol.2017.4 PMC5319707

[43] Tang Q , Jin G, Wang G, et al Current sampling methods for gut microbiota: a call for more precise devices. Front Cell Infect Microbiol. 2020;10:151. 32328469 10.3389/fcimb.2020.00151 PMC7161087

[44] Martinez JE , Kahana DD, Ghuman S, et al Unhealthy lifestyle and gut dysbiosis: a better understanding of the effects of poor diet and nicotine on the intestinal microbiome. Front Endocrinol (Lausanne). 2021;12:667066. 34168615 10.3389/fendo.2021.667066 PMC8218903

[45] Molodecky NA , Kaplan GG. Environmental risk factors for inflammatory bowel disease. Gastroenterol Hepatol (N Y). 2010;6:339-346. 20567592 PMC2886488

[46] Al Radi ZMA , Prins FM, Collij V, et al Exploring the predictive value of gut microbiome signatures for therapy intensification in patients with inflammatory bowel disease: a 10-year follow-up study. Inflamm Bowel Dis. 2024;30:1642-1653. 38635882 10.1093/ibd/izae064 PMC11446998

[47] Ananthakrishnan AN , Luo C, Yajnik V, et al Gut microbiome function predicts response to anti-integrin biologic therapy in inflammatory bowel diseases. Cell Host Microbe. 2017;21:603-610 e3. 28494241 10.1016/j.chom.2017.04.010 PMC5705050

[48] Huda-Faujan N , Abdulamir AS, Fatimah AB, et al The impact of the level of the intestinal short chain fatty acids in inflammatory bowel disease patients versus healthy subjects. Open Biochem J. 2010;4:53-58. 20563285 10.2174/1874091X01004010053 PMC2887640

[49] Machiels K , Joossens M, Sabino J, et al A decrease of the butyrate-producing species *Roseburia hominis* and *Faecalibacterium prausnitzii* defines dysbiosis in patients with ulcerative colitis. Gut. 2014;63:1275-1283. 24021287 10.1136/gutjnl-2013-304833

[50] Ding NS , McDonald JAK, Perdones-Montero A, et al Metabonomics and the gut microbiome associated with primary response to anti-TNF therapy in Crohn’s disease. J Crohns Colitis. 2020;14:1090-1102. 32119090 10.1093/ecco-jcc/jjaa039

[51] Eslami M , Naderian R, Bahar A, et al Microbiota as diagnostic biomarkers: advancing early cancer detection and personalized therapeutic approaches through microbiome profiling. Front Immunol. 2025;16:1559480. 40406094 10.3389/fimmu.2025.1559480 PMC12095362

[52] Loyfer N , Magenheim J, Peretz A, et al A DNA methylation atlas of normal human cell types. Nature. 2023;613:355-364. 36599988 10.1038/s41586-022-05580-6 PMC9811898

[53] Liu H , Zhou J, Tian W, et al DNA methylation atlas of the mouse brain at single-cell resolution. Nature. 2021;598:120-128. 34616061 10.1038/s41586-020-03182-8 PMC8494641

[54] Horvath S , Raj K. DNA methylation-based biomarkers and the epigenetic clock theory of ageing. Nat Rev Genet. 2018;19:371-384. 29643443 10.1038/s41576-018-0004-3

[55] Feil R , Fraga MF. Epigenetics and the environment: emerging patterns and implications. Nat Rev Genet. 2012;13:97-109. 22215131 10.1038/nrg3142

[56] Onieva-Garcia MA , Llanos-Mendez A, Banos-Alvarez E, Isabel-Gomez R. A systematic review of the clinical validity of the Cologuard genetic test for screening colorectal cancer. Rev Clin Esp (Barc). 2015;215:527-536. 26434810 10.1016/j.rce.2015.08.002

[57] Lamb YN , Dhillon S. Epi proColon((R)) 2.0 CE: a blood-based screening test for colorectal cancer. Mol Diagn Ther. 2017;21:225-232. 28155091 10.1007/s40291-017-0259-y

[58] Everhard S , Tost J, El Abdalaoui H, et al Identification of regions correlating MGMT promoter methylation and gene expression in glioblastomas. Neuro Oncol. 2009;11:348-356. 19224763 10.1215/15228517-2009-001 PMC2743215

[59] Malley DS , Hamoudi RA, Kocialkowski S, Pearson DM, Collins VP, Ichimura K. A distinct region of the MGMT CpG island critical for transcriptional regulation is preferentially methylated in glioblastoma cells and xenografts. Acta Neuropathol. 2011;121:651-661. 21287394 10.1007/s00401-011-0803-5

[60] Hegi ME , Genbrugge E, Gorlia T, et al MGMT promoter methylation cutoff with safety margin for selecting glioblastoma patients into trials omitting temozolomide: a pooled analysis of four clinical trials. Clin Cancer Res. 2019;25:1809-1816. 30514777 10.1158/1078-0432.CCR-18-3181 PMC8127866

[61] Waterhouse RL , Van Neste L, Moses KA, et al Evaluation of an epigenetic assay for predicting repeat prostate biopsy outcome in African American men. Urology. 2019;128:62-65. 29660369 10.1016/j.urology.2018.04.001 PMC10182891

[62] Partin AW , Van Neste L, Klein EA, et al Clinical validation of an epigenetic assay to predict negative histopathological results in repeat prostate biopsies. J Urol. 2014;192:1081-1087. 24747657 10.1016/j.juro.2014.04.013 PMC4337855

[63] Stewart GD , Van Neste L, Delvenne P, et al Clinical utility of an epigenetic assay to detect occult prostate cancer in histopathologically negative biopsies: results of the MATLOC study. J Urol. 2013;189:1110-1116. 22999998 10.1016/j.juro.2012.08.219

[64] Sill M , Schrimpf D, Patel A, et al Advancing CNS tumor diagnostics with expanded DNA methylation-based classification. Cancer Cell. 2026;44:340-354.e2. 41349541 10.1016/j.ccell.2025.11.002

[65] Liu Y , Aryee MJ, Padyukov L, et al Epigenome-wide association data implicate DNA methylation as an intermediary of genetic risk in rheumatoid arthritis. Nat Biotechnol. 2013;31:142-147. 23334450 10.1038/nbt.2487 PMC3598632

[66] Wang J , Dang X, Wu X, et al DNA methylation of IFI44L as a potential blood biomarker for childhood-onset systemic lupus erythematosus. Pediatr Res. 2024;96:494-501. 38514858 10.1038/s41390-024-03135-1 PMC11343705

[67] Zhao M , Zhou Y, Zhu B, et al IFI44L promoter methylation as a blood biomarker for systemic lupus erythematosus. Ann Rheum Dis. 2016;75:1998-2006. 26787370 10.1136/annrheumdis-2015-208410 PMC4955646

[68] Chen Z , Miao F, Braffett BH, et al; DCCT/EDIC Study Group. DNA methylation mediates development of HbA1c-associated complications in type 1 diabetes. Nat Metab. 2020;2:744-762. 32694834 10.1038/s42255-020-0231-8 PMC7590966

[69] Adams AT , Kennedy NA, Hansen R, et al Two-stage genome-wide methylation profiling in childhood-onset Crohn’s disease implicates epigenetic alterations at the VMP1/MIR21 and HLA loci. Inflamm Bowel Dis. 2014;20:1784-1793. 25144570 10.1097/MIB.0000000000000179 PMC4736293

[70] McDermott E , Ryan EJ, Tosetto M, et al DNA methylation profiling in inflammatory bowel disease provides new insights into disease pathogenesis. J Crohns Colitis. 2016;10:77-86. 26419460 10.1093/ecco-jcc/jjv176 PMC5013897

[71] Ventham NT , Kennedy NA, Adams AT, et al; IBD CHARACTER consortium. Integrative epigenome-wide analysis demonstrates that DNA methylation may mediate genetic risk in inflammatory bowel disease. Nat Commun. 2016;7:13507. 27886173 10.1038/ncomms13507 PMC5133631

[72] Somineni HK , Venkateswaran S, Kilaru V, et al Blood-derived DNA methylation signatures of Crohn’s disease and severity of intestinal inflammation. Gastroenterology. 2019;156:2254-2265 e3. 30779925 10.1053/j.gastro.2019.01.270 PMC6529254

[73] Mishra N , Aden K, Blase JI, et al; SYSCID Consortium. Longitudinal multi-omics analysis identifies early blood-based predictors of anti-TNF therapy response in inflammatory bowel disease. Genome Med. 2022;14:110. 36153599 10.1186/s13073-022-01112-z PMC9509553

[74] Joustra V , Li Yim AYF, Hageman I, et al Long-term temporal stability of peripheral blood DNA methylation profiles in patients with inflammatory bowel disease. Cell Mol Gastroenterol Hepatol. 2023;15:869-885. 36581079 10.1016/j.jcmgh.2022.12.011 PMC9972576

[75] Lin S , Hannon E, Reppell M, et al Whole blood DNA methylation changes are associated with anti-TNF drug concentration in patients with Crohn’s disease. J Crohns Colitis. 2024;18:1190-1201. 37551994 10.1093/ecco-jcc/jjad133 PMC11324340

[76] Noble A , Adams A, Nowak J, et al The circulating methylome in childhood-onset inflammatory bowel disease. J Crohns Colitis. 2025;19:jjae157. 39365013 10.1093/ecco-jcc/jjae157 PMC11945304

[77] Joustra VW , Li Yim AYF, Henneman P, et al; EPIC-CD Consortium. Development and validation of peripheral blood DNA methylation signatures to predict response to biological therapy in adults with Crohn’s disease (EPIC-CD): an epigenome-wide association study. Lancet Gastroenterol Hepatol. 2025;10:818-830. 40614748 10.1016/S2468-1253(25)00102-5

[78] Harris RA , Nagy-Szakal D, Mir SAV, et al DNA methylation-associated colonic mucosal immune and defense responses in treatment-naïve pediatric ulcerative colitis. Epigenetics. 2014;9:1131-1137. 24937444 10.4161/epi.29446 PMC4164498

[79] Howell KJ , Kraiczy J, Nayak KM, et al DNA methylation and transcription patterns in intestinal epithelial cells from pediatric patients with inflammatory bowel diseases differentiate disease subtypes and associate with outcome. Gastroenterology. 2018;154:585-598. 29031501 10.1053/j.gastro.2017.10.007 PMC6381389

[80] Agliata I , Fernandez-Jimenez N, Goldsmith C, et al The DNA methylome of inflammatory bowel disease (IBD) reflects intrinsic and extrinsic factors in intestinal mucosal cells. Epigenetics. 2020;15:1068-1082. 32281463 10.1080/15592294.2020.1748916 PMC7518701

[81] Li Y , Wang Z, Wu X, et al Intestinal mucosa-derived DNA methylation signatures in the penetrating intestinal mucosal lesions of Crohn’s disease. Sci Rep. 2021;11:9771. 33963246 10.1038/s41598-021-89087-6 PMC8105344

[82] Edgar RD , Perrone F, Foster AR, et al Culture-associated DNA methylation changes impact on cellular function of human intestinal organoids. Cell Mol Gastroenterol Hepatol. 2022;14:1295-1310. 36038072 10.1016/j.jcmgh.2022.08.008 PMC9703134

[83] Anders S , Huber W. Differential expression analysis for sequence count data. Genome Biol. 2010;11:R106. 20979621 10.1186/gb-2010-11-10-r106 PMC3218662

[84] Xie C , Leung YK, Chen A, Long DX, Hoyo C, Ho SM. Differential methylation values in differential methylation analysis. Bioinformatics. 2019;35:1094-1097. 30184051 10.1093/bioinformatics/bty778 PMC6449748

[85] Tripepi G , Jager KJ, Dekker FW, Zoccali C. Linear and logistic regression analysis. Kidney Int. 2008;73:806-810. 18200004 10.1038/sj.ki.5002787

[86] Flynn R. Survival analysis. J Clin Nurs. 2012;21:2789-2797. 22860755 10.1111/j.1365-2702.2011.04023.x

[87] Langfelder P , Horvath S. WGCNA: an R package for weighted correlation network analysis. BMC Bioinformatics. 2008;9:559. 19114008 10.1186/1471-2105-9-559 PMC2631488

[88] Pai S , Bader GD. Patient similarity networks for precision medicine. J Mol Biol. 2018;430:2924-2938. 29860027 10.1016/j.jmb.2018.05.037 PMC6097926

[89] Suo Q , Ma F, Yuan Y, et al Deep patient similarity learning for personalized healthcare. IEEE Trans Nanobioscience. 2018;17:219-227. 29994534 10.1109/TNB.2018.2837622

[90] Huynh-Thu VA , Saeys Y, Wehenkel L, Geurts P. Statistical interpretation of machine learning-based feature importance scores for biomarker discovery. Bioinformatics. 2012;28:1766-1774. 22539669 10.1093/bioinformatics/bts238

[91] Ng S , Masarone S, Watson D, Barnes MR. The benefits and pitfalls of machine learning for biomarker discovery. Cell Tissue Res. 2023;394:17-31. 37498390 10.1007/s00441-023-03816-z PMC10558383

[92] Zhang Z , Zhao Y, Liao X, et al Deep learning in omics: a survey and guideline. Brief Funct Genomics. 2019;18:41-57. 30265280 10.1093/bfgp/ely030

[93] Janiesch C , Zschech P, Heinrich K. Machine learning and deep learning. Electron Markets. 2021;31:685-695.

[94] Guo F , Guan R, Li Y, et al Foundation models in bioinformatics. Natl Sci Rev. 2025;12:nwaf028. 40078374 10.1093/nsr/nwaf028 PMC11900445

[95] Kosorok MR , Ma S. Marginal asymptotics for the “large p, small n” paradigm: With applications to microarray data. Ann Statist. 2007;35:1456-1486.

[96] Abdi H , Williams LJ. Principal component analysis. WIREs Computational Stats. 2010;2:433-459.

[97] Healy J , Mclnnes L. Uniform manifold approximation and projection. *Nat Rev Methods Primers*. 2024;4:82.

[98] Lvd M , Hinton G. Visualizing data using t-SNE. J Machine Learn Res. 2008;9:2579-2605.

[99] Jia WK , Sun ML, Lian J, Hou SJ. Feature dimensionality reduction: a review. Complex Intell Syst. 2022;8:2663-2693.

[100] Islam MT , Xing L. A data-driven dimensionality-reduction algorithm for the exploration of patterns in biomedical data. Nat Biomed Eng. 2021;5:624-635. 33139824 10.1038/s41551-020-00635-3

[101] Lichou F , Orazio S, Dulucq S, et al Novel analytical methods to interpret large sequencing data from small sample sizes. Hum Genomics. 2019;13:41. 31470908 10.1186/s40246-019-0235-1 PMC6717342

[102] Dwivedi AK , Mallawaarachchi I, Alvarado LA. Analysis of small sample size studies using nonparametric bootstrap test with pooled resampling method. Stat Med. 2017;36:2187-2205. 28276584 10.1002/sim.7263

[103] Zheng Y , Keleş S. FreeHi-C simulates high-fidelity Hi-C data for benchmarking and data augmentation. Nat Methods. 2020;17:37-40. 31712779 10.1038/s41592-019-0624-3 PMC8136837

[104] Calimeri F , Marzullo A, Stamile C, Terracina G. Biomedical data augmentation using generative adversarial neural networks. In: Tetko IV, Kurkova V, Karpov P, Theis F (eds.), International Conference on Artificial Neural Networks. Springer; 2017.

[105] de Lima Camillo LP , Sehgal R, Armstrong J, et al CpGPT: a foundation model for DNA methylation. bioRxiv, 2024:2024.10. 24.619766, preprint: not peer reviewed.

[106] Ying K , Song J, Cui H, et al MethylGPT: a foundation model for the DNA methylome. bioRxiv, 2024, preprint: not peer reviewed.

[107] Velten B , Braunger JM, Argelaguet R, et al Identifying temporal and spatial patterns of variation from multimodal data using MEFISTO. Nat Methods. 2022;19:179-186. 35027765 10.1038/s41592-021-01343-9 PMC8828471

[108] Jain S , Safo SE. DeepIDA-GRU: a deep learning pipeline for integrative discriminant analysis of cross-sectional and longitudinal multiview data with applications to inflammatory bowel disease classification. Brief Bioinform. 2024;25:bbae339. 39007595 10.1093/bib/bbae339 PMC11771283

[109] Bossa MN , Sahli H. A multidimensional ODE-based model of Alzheimer’s disease progression. Sci Rep. 2023;13:3162. 36823416 10.1038/s41598-023-29383-5 PMC9950424

[110] Kilian C , Ulrich H, Zouboulis VA, et al Longitudinal single-cell data informs deterministic modelling of inflammatory bowel disease. NPJ Syst Biol Appl. 2024;10:69. 38914538 10.1038/s41540-024-00395-9 PMC11196733

[111] Yi H-C , You Z-H, Huang D-S, Kwoh CK. Graph representation learning in bioinformatics: trends, methods and applications. Brief Bioinform. 2022;23:bbab340. 34471921 10.1093/bib/bbab340

[112] Li MM , Huang K, Zitnik M. Graph representation learning in biomedicine and healthcare. Nat Biomed Eng. 2022;6:1353-1369. 36316368 10.1038/s41551-022-00942-x PMC10699434

[113] Murtagh F , Contreras P. Algorithms for hierarchical clustering: an overview. WIREs Data Min & Knowl. 2012;2:86-97.

[114] Wang B , Mezlini AM, Demir F, et al Similarity network fusion for aggregating data types on a genomic scale. Nat Methods. 2014;11:333-337. 24464287 10.1038/nmeth.2810

[115] Wang T , Shao W, Huang Z, et al MOGONET integrates multi-omics data using graph convolutional networks allowing patient classification and biomarker identification. Nat Commun. 2021;12:3445. 34103512 10.1038/s41467-021-23774-w PMC8187432

[116] Wilkinson C , Bhatty A, Batra G, et al; Global Cardiovascular Outcomes Consortium and in collaboration with ACNAP, ACVC, EACVI, EAPC, EAPCI, EHRA, ESC Committee for Young CV Professionals, ESC Registry Committee, HFA, ESC Patient Forum and these Working Groups: aorta and peripheral vascular diseases, atherosclerosis and vascular biology, cardiac cellular electrophysiology, cardiovascular pharmacotherapy, cardiovascular regenerative and restorative medicine, cardiovascular surgery, cellular biology of the heart, e-cardiology, myocardial function, pulmonary circulation and right ventricular function and thrombosis. Definitions of clinical study outcome measures for cardiovascular diseases: the European unified registries for heart care evaluation and randomized trials (EuroHeart). Eur Heart J. 2025;46:190-214. 39545867 10.1093/eurheartj/ehae724 PMC11704390

[117] Jammer I , Wickboldt N, Sander M, et al; European Society of Intensive Care Medicine. Standards for definitions and use of outcome measures for clinical effectiveness research in perioperative medicine: European Perioperative Clinical Outcome (EPCO) definitions: a statement from the ESA-ESICM joint taskforce on perioperative outcome measures. Eur J Anaesthesiol. 2015;32:88-105. 25058504 10.1097/EJA.0000000000000118

[118] Malhotra A , Ayappa I, Ayas N, et al Metrics of sleep apnea severity: beyond the apnea-hypopnea index. Sleep. 2021;44:zsab030. 33693939 10.1093/sleep/zsab030 PMC8271129

[119] Ylescupidez A , Bahnson HT, O’Rourke C, Lord S, Speake C, Greenbaum CJ. A standardized metric to enhance clinical trial design and outcome interpretation in type 1 diabetes. Nat Commun. 2023;14:7214. 37940642 10.1038/s41467-023-42581-z PMC10632453

[120] Reps JM , Schuemie MJ, Suchard MA, Ryan PB, Rijnbeek PR. Design and implementation of a standardized framework to generate and evaluate patient-level prediction models using observational healthcare data. J Am Med Inform Assoc. 2018;25:969-975. 29718407 10.1093/jamia/ocy032 PMC6077830

[121] Morales J , Welter D, Bowler EH, et al A standardized framework for representation of ancestry data in genomics studies, with application to the NHGRI-EBI GWAS Catalog. Genome Biol. 2018;19:21. 29448949 10.1186/s13059-018-1396-2 PMC5815218

[122] Wan Q , Tang J, Han Y, Wang D. Co-expression modules construction by WGCNA and identify potential prognostic markers of uveal melanoma. Exp Eye Res. 2018;166:13-20. 29031853 10.1016/j.exer.2017.10.007

[123] Zhai X , Xue Q, Liu Q, Guo Y, Chen Z. Colon cancer recurrence‑associated genes revealed by WGCNA co‑expression network analysis. Mol Med Rep. 2017;16:6499-6505. 28901407 10.3892/mmr.2017.7412 PMC5865817

[124] Levy JJ , Titus AJ, Petersen CL, Chen Y, Salas LA, Christensen BC. MethylNet: an automated and modular deep learning approach for DNA methylation analysis. BMC Bioinformatics. 2020;21:108. 32183722 10.1186/s12859-020-3443-8 PMC7076991

[125] Lee Y , Cappellato M, Di Camillo B. Machine learning-based feature selection to search stable microbial biomarkers: application to inflammatory bowel disease. Gigascience. 2022;12:giad083. 37882604 10.1093/gigascience/giad083 PMC10600917

[126] Cao ZJ , Gao G. Multi-omics single-cell data integration and regulatory inference with graph-linked embedding. Nat Biotechnol. 2022;40:1458-1466. 35501393 10.1038/s41587-022-01284-4 PMC9546775

[127] Lan W , Liao H, Chen Q, Zhu L, Pan Y, Chen YP. DeepKEGG: a multi-omics data integration framework with biological insights for cancer recurrence prediction and biomarker discovery. Brief Bioinform. 2024;25:bbae185. 38678587 10.1093/bib/bbae185 PMC11056029

[128] Haghverdi L , Lun ATL, Morgan MD, Marioni JC. Batch effects in single-cell RNA-sequencing data are corrected by matching mutual nearest neighbors. Nat Biotechnol. 2018;36:421-427. 29608177 10.1038/nbt.4091 PMC6152897

[129] Korsunsky I , Millard N, Fan J, et al Fast, sensitive and accurate integration of single-cell data with Harmony. Nat Methods. 2019;16:1289-1296. 31740819 10.1038/s41592-019-0619-0 PMC6884693

[130] Lopez R , Regier J, Cole MB, Jordan MI, Yosef N. Deep generative modeling for single-cell transcriptomics. Nat Methods. 2018;15:1053-1058. 30504886 10.1038/s41592-018-0229-2 PMC6289068

[131] Zhang Y , Parmigiani G, Johnson WE. ComBat-seq: batch effect adjustment for RNA-seq count data. NAR Genom Bioinform. 2020;2:lqaa078. 33015620 10.1093/nargab/lqaa078 PMC7518324

[132] Hrovatin K , Sikkema L, Shitov VA, et al Considerations for building and using integrated single-cell atlases. Nat Methods. 2025;22:41-57. 39672979 10.1038/s41592-024-02532-y

[133] Cho J-W , Shim HS, Lee CY, et al The importance of enhancer methylation for epigenetic regulation of tumorigenesis in squamous lung cancer. Exp Mol Med. 2022;54:12-22. 34987166 10.1038/s12276-021-00718-4 PMC8813945

[134] Heyn H , Vidal E, Ferreira HJ, et al Epigenomic analysis detects aberrant super-enhancer DNA methylation in human cancer. Genome Biol. 2016;17:11. 26813288 10.1186/s13059-016-0879-2 PMC4728783

[135] Silva TC , Coetzee SG, Gull N, et al ELMER v. 2: an R/Bioconductor package to reconstruct gene regulatory networks from DNA methylation and transcriptome profiles. Bioinformatics. 2019;35:1974-1977. 30364927 10.1093/bioinformatics/bty902 PMC6546131

[136] Tong Y , Sun J, Wong CF, et al MICMIC: identification of DNA methylation of distal regulatory regions with causal effects on tumorigenesis. Genome Biol. 2018;19:73. 29871649 10.1186/s13059-018-1442-0 PMC5989391

[137] Fulco CP , Nasser J, Jones TR, et al Activity-by-contact model of enhancer–promoter regulation from thousands of CRISPR perturbations. Nat Genet. 2019;51:1664-1669. 31784727 10.1038/s41588-019-0538-0 PMC6886585

[138] Maksimovic J , Oshlack A, Phipson B. Gene set enrichment analysis for genome-wide DNA methylation data. Genome Biol. 2021;22:173. 34103055 10.1186/s13059-021-02388-x PMC8186068

[139] Itai Y , Rappoport N, Shamir R. Integration of gene expression and DNA methylation data across different experiments. Nucleic Acids Res. 2023;51:7762-7776. 37395437 10.1093/nar/gkad566 PMC10450176

[140] Fleischer T , Tekpli X, Mathelier A, et al; Oslo Breast Cancer Research Consortium (OSBREAC). DNA methylation at enhancers identifies distinct breast cancer lineages. Nat Commun. 2017;8:1379. 29123100 10.1038/s41467-017-00510-x PMC5680222

[141] Yang J , Wang Q, Zhang Z-Y, et al DNA methylation-based epigenetic signatures predict somatic genomic alterations in gliomas. Nat Commun. 2022;13:4410. 35906213 10.1038/s41467-022-31827-x PMC9338285

[142] Xie J , Song Y, Zheng H, et al PathMethy: an interpretable AI framework for cancer origin tracing based on DNA methylation. Brief Bioinform. 2024;25:bbae497. 39391931 10.1093/bib/bbae497 PMC11467402

[143] Haghani A , Li CZ, Robeck TR, et al DNA methylation networks underlying mammalian traits. Science. 2023;381:eabq5693. 37561875 10.1126/science.abq5693 PMC11180965

[144] Mohr AE , Ortega-Santos CP, Whisner CM, Klein-Seetharaman J, Jasbi P. Navigating challenges and opportunities in multi-omics integration for personalized healthcare. Biomedicines. 2024;12:1496. 39062068 10.3390/biomedicines12071496 PMC11274472

[145] Argelaguet R , Velten B, Arnol D, et al Multi-omics factor analysis-a framework for unsupervised integration of multi-omics data sets. Mol Syst Biol. 2018;14:e8124. 29925568 10.15252/msb.20178124 PMC6010767

[146] Pekayvaz K , Losert C, Knottenberg V, et al Multiomic analyses uncover immunological signatures in acute and chronic coronary syndromes. Nat Med. 2024;30:1696-1710. 38773340 10.1038/s41591-024-02953-4 PMC11186793

[147] Clark C , Dayon L, Masoodi M, Bowman GL, Popp J. An integrative multi-omics approach reveals new central nervous system pathway alterations in Alzheimer’s disease. Alz Res Therapy. 2021;13:71. 10.1186/s13195-021-00814-7 PMC8015070 33794997

[148] Yang M , Matan-Lithwick S, Wang Y, De Jager PL, Bennett DA, Felsky D. Multi-omic integration via similarity network fusion to detect molecular subtypes of ageing. Brain Commun. 2023;5:fcad110. 37082508 10.1093/braincomms/fcad110 PMC10110975

[149] Ma S , Zeng AG, Haibe-Kains B, Goldenberg A, Dick JE, Wang B. Moving towards genome-wide data integration for patient stratification with integrate any omics. Nat Mach Intell. 2025;7:29-42.

[150] Shen R , Olshen AB, Ladanyi M. Integrative clustering of multiple genomic data types using a joint latent variable model with application to breast and lung cancer subtype analysis. Bioinformatics. 2009;25:2906-2912. 19759197 10.1093/bioinformatics/btp543 PMC2800366

[151] Bibikova M , Le J, Barnes B, et al Genome-wide DNA methylation profiling using Infinium^®^ assay. Epigenomics. 2009;1:177-200. 22122642 10.2217/epi.09.14

[152] Meissner A , Mikkelsen TS, Gu H, et al Genome-scale DNA methylation maps of pluripotent and differentiated cells. Nature. 2008;454:766-770. 18600261 10.1038/nature07107 PMC2896277

[153] Cokus SJ , Feng S, Zhang X, et al Shotgun bisulphite sequencing of the Arabidopsis genome reveals DNA methylation patterning. Nature. 2008;452:215-219. 18278030 10.1038/nature06745 PMC2377394

[154] Zhou W , Triche TJ Jr, Laird PW, Shen H. SeSAMe: reducing artifactual detection of DNA methylation by Infinium BeadChips in genomic deletions. Nucleic Acids Res. 2018;46:e123-e. 30085201 10.1093/nar/gky691 PMC6237738

[155] Aryee MJ , Jaffe AE, Corrada-Bravo H, et al Minfi: a flexible and comprehensive Bioconductor package for the analysis of Infinium DNA methylation microarrays. Bioinformatics. 2014;30:1363-1369. 24478339 10.1093/bioinformatics/btu049 PMC4016708

[156] Zou LS , Erdos MR, Taylor DL, Chines PS, Varshney A, et al; edu MGIrw. BoostMe accurately predicts DNA methylation values in whole-genome bisulfite sequencing of multiple human tissues. BMC Genomics. 2018;19:390. 29792182 10.1186/s12864-018-4766-y PMC5966887

[157] Taudt A , Roquis D, Vidalis A, Wardenaar R, Johannes F, Colomé-Tatché M. METHimpute: imputation-guided construction of complete methylomes from WGBS data. BMC Genomics. 2018;19:444. 29879918 10.1186/s12864-018-4641-x PMC5992726

[158] Houseman EA , Accomando WP, Koestler DC, et al DNA methylation arrays as surrogate measures of cell mixture distribution. BMC Bioinformatics. 2012;13:86. 22568884 10.1186/1471-2105-13-86 PMC3532182

[159] Teschendorff AE , Breeze CE, Zheng SC, Beck S. A comparison of reference-based algorithms for correcting cell-type heterogeneity in epigenome-wide association studies. BMC Bioinformatics. 2017;18:105. 28193155 10.1186/s12859-017-1511-5 PMC5307731

[160] Zhang W , Wu H, Li Z. Complete deconvolution of DNA methylation signals from complex tissues: a geometric approach. Bioinformatics. 2021;37:1052-1059. 33135072 10.1093/bioinformatics/btaa930 PMC8150138

[161] Scherer M , Nazarov PV, Toth R, et al Reference-free deconvolution, visualization and interpretation of complex DNA methylation data using DecompPipeline, MeDeCom and FactorViz. Nat Protoc. 2020;15:3240-3263. 32978601 10.1038/s41596-020-0369-6

[162] Teschendorff AE , Zhu T, Breeze CE, Beck S. EPISCORE: cell type deconvolution of bulk tissue DNA methylomes from single-cell RNA-Seq data. Genome Biol. 2020;21:221. 32883324 10.1186/s13059-020-02126-9 PMC7650528

